# Axon Initial Segment: Structure, Biological Functions, Diseases, and Therapeutic Targets

**DOI:** 10.1002/mco2.70689

**Published:** 2026-03-19

**Authors:** Dong‐Yan Song, Lin Yuan, Weiguo Yang, Wen Li, Jia‐Yi Li

**Affiliations:** ^1^ Laboratory of Research in Parkinson's Disease and Related Disorders Health Sciences Institute Key Laboratory of Major Chronic Diseases of Nervous System of Liaoning Province China Medical University Shenyang China; ^2^ Department of Neurobiology Key Laboratory of Cell Biology Ministry of Public Health and Key Laboratory of Medical Cell Biology Ministry of Education China Medical University Shenyang China; ^3^ Neural Plasticity and Repair Unit Department of Experimental Medical Sciences Lund University Lund Sweden

**Keywords:** axon initial segment, action potential, neural circuit, neurological disorders, plasticity

## Abstract

The axon initial segment (AIS) is a specialized neuronal microdomain that serves as a physical diffusion barrier, separating the axon from somatodendritic compartments. As a highly plastic structure, the AIS dynamically regulates neuronal excitability and contributes to circuit homeostasis. Recent advances in super‐resolution imaging and disease modeling have expanded our understanding of its role in neurodevelopment and neurodegenerative disorders. This review first systematically outlines the molecular architecture of the AIS, including its cytoskeletal scaffolds and ion‐channel complexes. Then, we discuss AIS plasticity, ranging from activity‐dependent alterations to the molecular mechanisms that regulate it, and to its key biological functions, such as its role in action potential initiation, neuronal polarization, subcellular organelle sorting, and neural circuit excitability. We further highlight emerging evidence that AIS disruption represents an early pathological event in neurodegenerative and neuropsychiatric disorders. By integrating physiological and pathological perspectives, and by evaluating emerging biomarker strategies and therapeutic interventions, this review outlines directions and challenges for future AIS‐targeted therapies. Meanwhile, it summarizes key experimental and potential clinical tools for future AIS research. Overall, elucidating the molecular mechanism of the AIS in both health and disease provides a deeper understanding for advancing the diagnosis and treatment of neurological diseases.

## Graphical abstract

The axon initial segment (AIS) is a specialized axonal domain critical for maintaining neuronal polarity, action potential initiation, and network stability. This review summarizes the necessary elements for maintaining AIS structure and plasticity, discussing crucial biological functions of AIS in neuronal signaling and the neural circuit homeostasis, and explores the mechanism of abnormal AIS participating in various neurological disorders. By integrating physiological and pathological perspectives, we propose the AIS as a promising therapeutic target for neurological diseases and discuss the development of experimental and clinical evaluation tools that facilitate in‐depth research into AIS.

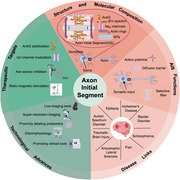

## 1 Introduction

The axon initial segment (AIS), a 20–60 µm proximal axonal domain first identified by electron microscopy in the 1960s [1, 2], serves as a critical hub for neuronal function. It acts not only as a diffusion barrier that maintains neuronal polarity, but also as the primary site for integrating synaptic inputs into action potentials (APs) [3, 4]. The AIS maintains neuronal polarity through two principal mechanisms: restricting the mobility of membrane components [5] and acting as an intracellular filter, preventing somatodendritic cargoes from entering the axon [6]. Molecular architecture of AIS—comprising ion channels (e.g., Na_V_, K_V_), scaffolding proteins (e.g., ankyrin G [AnkG]), and cytoskeletal elements (e.g., βIV‐spectrin, microtubules [MTs])—enables dynamic regulation of neuronal excitability [7].

Emerging evidence underscores the (patho‐)physiological significance of AIS plasticity [8]. The activity‐dependent remodeling of its molecular composition, length, and location is crucial for the homeostatic regulation of neuronal intrinsic excitability [9, 10]. Impaired plasticity of AIS is closely associated with aberrant neuronal excitability in Alzheimer's disease (AD), amyotrophic lateral sclerosis (ALS), and epilepsy [11, 12, 13]. Furthermore, disruptions in AIS integrity are implicated in the early stages of neurodevelopmental and psychiatric disorders such as autism spectrum disorder (ASD) and bipolar disorder (BD), which are often associated with mutations in AIS‐associated genes like *ANK3* (encoding AnkG) and *SCN2A* (encoding Na_V_1.2) [14, 15].

A focused review of the AIS is particularly timely, driven by recent technological breakthroughs. Super‐resolution imaging has unveiled its nanoscale architecture with unprecedented clarity [16, 17, 18], while novel in vivo reporter mouse models and innovative proteomics techniques have made progress in deciphering its dynamic physiology and composition [19]. These advances have profoundly deepened our understanding of the AIS's dual roles in health and disease, positioning it as a promising therapeutic target for neurological disorders.

This review begins by establishing a molecular foundation of the AIS, detailing the core complexes that support its structure and function. We then explore the mechanisms and modulators of its structural and functional plasticity, systematically examine its key biological roles in neuronal signaling and circuit homeostasis, and delineate its involvement across a broad spectrum of diseases. Finally, we evaluate potential therapeutic targets and strategies directed at the AIS, summarize advanced experimental and potential clinical assessment tools for its investigation, and discuss the challenges and future directions in translating AIS‐related basic research into clinical applications.

## 2 Structure and Molecular Composition

The complex and specialized molecular structure of AIS determines its precise physiological function. This section details the core molecular components of the AIS, including the key cytoskeletal scaffolds and organizers that define its structure, the voltage‐gated ion channels that regulate AP initiation, and other associated regulatory proteins critical for its function.

### 2.1 Key Cytoskeletal Components

AIS is assembled by a core protein complex orchestrated by AnkG [20]. The major molecular components encompass cytoskeletal scaffolds (mainly AnkG itself), voltage‐gated ion channels such as Na_V_, key cell adhesion molecules (notably the L1 family members neurofascin‐186 (NF‐186) and neuronal cell adhesion molecule (NrCAM)), protein kinases (such as casein kinase II (CK2)), extracellular matrix (ECM) constituents, and various regulatory accessory proteins [21, 22].

#### 2.1.1 Ankyrin G

The scaffolding protein AnkG is a pivotal organizer for AIS assembly and maintenance, as confirmed in pioneering studies by Bennett and colleagues [20, 23]. AnkG localizes explicitly at the AIS and nodes of Ranvier and exists primarily in two distinct isoforms, the 480 and 270 kDa [24]. AnkG comprises multiple functional domains, including an ankyrin repeat domain (responsible for membrane‐binding domain), a spectrin‐binding domain, a serine‐rich domain, and a tail domain [20]. AnkG uses its ankyrin repeat domain to directly interact with partners, including NrCAM, NF‐186, ion channels, and transporters, thereby anchoring them at the AIS [21]. It interacts with βIV‐spectrin and αII‐spectrin tetramers via its spectrin‐binding domain. The interaction forms a periodic cytoskeletal lattice that links membrane proteins to the actin cytoskeleton, thereby maintaining the structural integrity of the AIS [16, 25, 26, 27, 28, 29]. The neuro‐specific domain (also known as the serine‐rich domain or tail domain) interacts with multiple protein partners, including nuclear distribution element‐like 1 (Ndel1), GABA_A_ receptor‐associated protein (GABARAP), and MT end‐binding proteins [27, 30, 31, 32]. Mutations in, or reduced expression of, AnkG result in AIS disassembly, characterized by the loss of Na_V_ channels, K_V_7.2/7.3 channels, βIV‐spectrin, NF‐186 and NrCAM, as well as the absence of MT bundles in proximal axons [33, 34, 35, 36, 37, 38]. This disassembly consequently disrupts the cytoplasmic diffusion barrier and neuronal polarity, leading to the mis‐localization of somatodendritic proteins (e.g., MT‐associated protein 2 [MAP2]) into the proximal axon and even the ectopic acquisition of dendritic spines on the axonal membrane [6, 27, 33, 35]. Sobotzik et al. demonstrate that in cerebellar Purkinje cells of EGFP‐positive AnkG^−^/^−^ mice, nonspiny axons acquire a spiny phenotype within just 3 days [39]. These studies indicate that AnkG is indispensable for AIS assembly and crucial for the sustained maintenance of both neuronal polarity and structural integrity.

#### 2.1.2 Microtubules

At the ultrastructural level, the AIS is defined by three key features: tightly arranged MT bundles, an electron‐dense undercoating beneath the cytoplasmic membrane, and local ribosome clusters [1, 2]. These MT bundles, which form at the axon hillock and extend parallel throughout the AIS, serve as its principal identifying hallmarks [1, 2]. As core components of the inner AIS shaft, these MTs work in concert with actin filaments to play a pivotal role in axon specification and growth [27, 40]. Furthermore, this structure facilitates both long‐ and short‐range active axonal transport mechanisms that are essential for axonal function [40]. MTs bind to the carboxyterminal side of AnkG through MT plus‐end binding proteins EB1 and EB3, as well as MAPs, including Ndel1, a regulator of dynein activity [27, 30, 32]. The stabilization of MT bundles is also critical for maintaining AnkG localization, a process mediated by the cross‐linking factor 1 (MTCL1). Knockdown of MTCL1 in cerebellar Purkinje cells of mice disrupts the localization of AnkG, thereby compromising axonal polarity [41]. MT stability within the AIS also depends on the tripartite motif‐containing protein 46 (TRIM46). TRIM46 organizes MTs into tightly bundled, parallel arrays with uniformly oriented plus‐ends [42]. Dysfunction of TRIM46 impairs AnkG clustering, MT bundling, axon formation, and the precise sorting of vesicle transport [43]. Genetic ablation of MT tyrosinase and detyrosinase in murine models results in a pronounced reduction in AIS length, accompanied by an increased immobile fraction of endo‐lysosome in axons [44]. Additionally, retrograde axonal trafficking of endo‐lysosomes is significantly impaired under these conditions [44]. Beyond their structural role, MTs are integral to the tau‐diffusion‐barrier (TDB) at the AIS, where their dynamic regulation and stability are critical for anterograde sorting of tau and for maintaining TDB integrity [45]. These findings underscore the multifaceted contributions of MTs to AIS organization and function, spanning structural maintenance, organelle trafficking, and the compartmentalization of somatodendritic proteins.

#### 2.1.3 Tau

Tau is a neuronal MAP preferentially localized to axons [46]. The AIS composes a TDB that regulates retro‐ and anterograde trafficking of tau. Key AIS components, including AnkG, NrCAM, βIV‐spectrin, Na_V_, EB1/3, and glycogen synthase kinase‐3 (GSK‐3), collectively contribute to the retrograde TDB [47]. Pathological tau aggregates, such as the tau AD nucleation core (tau‐AC), impair AIS plasticity under conditions of chronic depolarization and aberrantly accumulate in somatodendritic compartments of primary hippocampal neurons [48]. Hyperphosphorylated tau further diminishes neuronal excitability by mediating distal relocalization of the AIS and destabilizing MT networks [49]. The frontotemporal dementia (FTD)‐linked V337M mutation of tau disrupts AIS cytoskeleton homeostasis, compromising both AIS plasticity and excitability homeostasis in induced pluripotent stem cells (iPSCs) from FTD patients [50]. Conversely, reduced tau expression attenuates activity‐dependent AIS plasticity in inhibitory neurons, yet enhances their excitatory and inhibitory output [51]. Notably, tau knockout in iPSC results in impaired AIS formation, underscoring its indispensable developmental role [52]. Collectively, these findings highlight tau as a central regulator of AIS plasticity, structural integrity, and neuronal excitability, with its dysregulation being a key mechanism in neurodegenerative diseases.

### 2.2 Ion Channels at the AIS

The AIS exhibits a cell‐type‐specific distribution of ion channels, allowing the output of individual neurons to be accurately and appropriately regulated. Dysregulation of ion channel expression and function at the AIS contributes to a spectrum of neurological disorders, particularly those involving neurodevelopmental deficits such as ASD [53] and epilepsy [54, 55]. Consequently, elucidating the spatial distribution and physiological roles of AIS‐localized ion channels is essential for deciphering the pathological mechanisms underlying various neuronal diseases (Table 1).

**TABLE 1:** Expression patterns of ion channels in the AIS and associated disorders.

Ion channel (gene name) at the AIS	Distribution	Function	Neuronal types	Associated disorders
Na_V_1.1 (*SCN1A*)	Predominantly at the proximal part of AIS	AP generation and propagation	GABAergic IN [54]; hippocampal CA3 PyN [56]; DRGN [57]; spinal cord MN [3]; RGC [56]	Epilepsy [54]; ASD [53]; neuropathic pain [57]
Na_V_1.2 (*SCN2A*)	Predominantly at the proximal part of AIS	AP generation and backpropagation; influence axonal and dendritic excitability; determine the somatodendritic potential threshold	Cortical and hippocampal CA1 PyN [55, 58]; SNc and VTA dopaminergic neurons [56]	Epilepsy [59]; schizophrenia [60]; BD [61]; ASD, ID [55]
Na_V_1.6 (*SCN8A*)	Predominantly at the distal part of AIS	AP initiation; determine the lowest threshold of AP initiation	Cortical and hippocampal CA1 and CA3 PyN [56]; RGC [3]; DGN [3]; cerebellar PC [56]; spinal cord MN [3]	ASD [15]
	Intense at the proximal part of AIS and reduced intensity at the distal part of AIS		GABAergic IN [56]	
Na_V_1.7 (*SCN9A*)	AIS enrichment	Spontaneous activity	DRGN [57]	Neuropathic pain [57]
Na_V_1.8 (*SCN10A*)	Colocalized with AnkG		Skin nerve fibers [62]	
Na_V_β4 (*SCN4B*)	AIS enrichment	Resurgent Na^+^ current; Persistent Na^+^ current; Repetitive firing	Cerebellar PC; subthalamic neurons; cortical PyN; cortical/hippocampal CA1/subiculum IN; spinal cord MN [63]	
K_V_1.1 (*KCNA1*)	At the distal part of AIS	AP waveform; AP repolarization; spiking pattern	Cortical and hippocampal CA1 and CA3 PyN [56]; GABAergic IN [56]; cerebellar stellate cell [3]; nucleus magnocellularis [64]; MNTB [3]	Episodic ataxia type 1 [65]; epilepsy [65]
K_V_1.2 (*KCNA2*)	At the distal part of AIS	AP waveform; AP repolarization; spiking pattern	RGC [56]; cortical and hippocampal CA1 and CA3 PyN [56]; GABAergic IN [56]; Cerebellar stellate cell [3]; MNTB [3]	
K_V_1.4 (*KCNA4*)	Enriched at the AIS	AP waveform	PyN [3]	
K_V_2.1 (*KCNB1*)	Restricted and clustered localization in the AIS	AP firing; neuron excitability	PyN [66]	Epilepsy [66]; schizophrenia [66]; BD [61]
K_V_2.2 (*KCNB2*)	Highly expressed in AIS	Maintain high frequency AP firing	MNTB [3, 67]	
K_V_3.1 (*KCNC1*)	Expressed in AIS, but more intense in the axon hillock	Homeostatic control of neuronal output	Nucleus magnocellularis [64]	Schizophrenia [68]
K_V_7.2 (*KCNQ2*)	At the distal part of AIS	AP generation and properties; set the resting membrane potential and regulate the firing threshold and frequency; M current	Nucleus magnocellularis [64]; cortical and hippocampal PyN [3]; spinal cord MN [3]; DGN [3]; cerebellar PC [3]; sciatic nerve [3]	Epilepsy [69]
K_V_7.3 (*KCNQ3*)	At the distal part of AIS	AP generation and properties; set the resting membrane potential and regulate the firing threshold and frequency; M current	Sciatic nerve [3]; cortical and hippocampal PyN [3]; spinal cord MN [3]; cerebellar PC [3]; DGN [3]	ASD [70]; BD [71]
K_Ca_2.3 (*KCNN3*)	Distribute uniformly in the AIS		Cortical and hippocampal PyN [72]	
K_2P_4.1 (*KCNK4/TRAAK*)	Distribute uniformly in the AIS	Neuronal excitability	Cortical and hippocampal PyN [73]	
Ca_V_2.1 (*CACNA1A*)	Interspersed in the AIS	Calcium influx; AP waveform; spike initiation; membrane potentials	Cortical PyN [3]; cerebellar PC [3]	Epilepsy and cerebellar ataxia [65]
Ca_V_2.2 (*CACNA1B*)	Interspersed in the AIS	Calcium influx; AP waveform; spike initiation; membrane potentials	Cortical PyN [3]; cerebellar PC [3]	BD [74]
Ca_V_2.3 (*CACNA1E*)	Interspersed in the AIS	Calcium influx; spiking and spike timing; the generation, shaping, and timing of AP bursts	Auditory brainstem cartwheel interneurons [75]; cortical PyN [75]; cerebellar PC [3]	
Ca_V_3.1 (*CACNA1G*)	Interspersed in the AIS	The threshold and timing of APs; calcium influx; spiking and spike timing; the generation, shaping, and timing of AP bursts	Auditory brainstem cartwheel interneurons [75]; cortical PyN [75]; cerebellar PC [75]	
Ca_V_3.2 (*CACNA1H*)	Interspersed in the AIS	The threshold and timing of APs; calcium influx; spiking and spike timing; the generation, shaping and timing of AP bursts	Auditory brainstem cartwheel interneurons [75]; cortical PyN [75]; cerebellar PC [75]	Epilepsy [65]; ASD [53]
HCN1	Low densities at the AIS	Spike probability; AP threshold	Principal neurons of the MSO [76]	Epilepsy [65]

Abbreviations: AIS, axon initial segment; AP, action potential; ASD, autism spectrum disorder; BD, bipolar disorder; DGN, dentate granule neuron; DRGN, dorsal root ganglion neurons; HCN, hyperpolarization‐activated cyclic nucleotide‐gated channels; ID, intellectual disability; IN, interneuron; MN, motor neuron; MNTB, medial nucleus of the trapezoid body; MSO, medial superior olive; PC, Purkinje cell; PyN, pyramidal neuron; RGC, retinal ganglion cell.

#### 2.2.1 Voltage‐Gated Sodium Channel (Na_V_)

The AIS is a specialized neuronal region where APs are initiated—a process that relies on a high density of Na_V_ channels. For instance, in the pyramidal neurons (PyNs) of cortical layer V, the density of Na_V_ channels at the AIS is approximately 50 times greater than in the soma and proximal dendrites [77]. Na_V_ channels are localized at the AIS via interaction with AnkG, mediated by a conserved motif within the intracellular loop between transmembrane domains II and III [78]. Furthermore, the phosphorylation of serine residues by CK2 enhances their affinity for AnkG, thereby promoting stabilization at these sites [79]. Multiple Na_V_ channel subtypes have been identified at the AIS, including Na_V_1.1, Na_V_1.2, Na_V_1.6, Na_V_1.7, Na_V_1.8, and Na_V_β4 [54, 57, 62, 63, 80, 81, 82]. Na_V_1.6, with its low activation threshold, is a critical subtype for AP initiation in most neurons and dominates in the mature AIS. In PyNs, both Na_V_1.2 and Na_V_1.6 are enriched at the AIS. However, during the development process, they exhibit distinct and dynamic localization patterns. At the early developmental stage, Na_V_1.2 is mainly distributed in the proximal AIS, dendrites, and unmyelinated distal axon branches, whereas Na_V_1.6 is concentrated at the distal AIS. As maturation and myelination proceed, Na_V_1.2 is excluded from the axon, and Na_V_1.6 becomes the dominant subtype at the AIS and nodes of Ranvier [83, 84]. A similar developmental shift occurs in retinal ganglion cells, where Na_V_1.2 is highly expressed at the AIS early on and is later replaced by Na_V_1.6 [85, 86, 87]. Na_V_1.1 is also enriched in the proximal AIS of retinal ganglion cells [85, 88]. In Purkinje cells, Na_V_1.6 is expressed at high density along the entire AIS [56]. Notably, a recent study identified Na_V_1.2 as the dominant subtype at the AIS of dopaminergic (DA) neurons in the substantia nigra (SN) and ventral tegmental area (VTA) of mice, with no detectable Na_V_1.1 or Na_V_1.6 immunosignals [89]. In contrast, Na_V_1.1 is primarily distributed at the proximal AIS of GABAergic interneurons [54, 90, 91], and is also present at the proximal AIS of spinal cord neurons—including about 80% of motor neurons—as well as in multiple brain regions of adult mice, such as the CA3 PyN layer of the hippocampus. Na_V_1.1 expression typically exhibits a gradient distribution from the proximal to the distal along the AIS. Its concentration is highest near the soma and gradually decreases toward the distal end, complementing the expression pattern of Na_V_1.6 [92]. Although Na_V_1.1 is detected in some PyNs, its expression level is much lower than the nearly ubiquitous expression in interneurons [92]. Na_V_β1 expression is relatively widespread at the AIS throughout the brain, particularly in the hippocampus, cortex, and cerebellum. Along most of the AIS, its distribution is uniform and is coexpressed with Na_V_1.1, Na_V_1.2, and Na_V_1.6 subunits in wild‐type mice. Although present in both excitatory and inhibitory AIS, Na_V_β1 exhibits significant fine heterogeneity, which may contribute to the regulation of neuronal and regional excitability [93].

Functional studies utilizing knockout models highlight the distinct contributions of these Na_V_ subtypes. Na_V_1.6 deficiency in PyNs reduces the noninactivated Na^+^ current, elevates the voltage threshold for AP initiation, and lowers transient firing rates [81]. Conversely, Na_V_1.2 deficiency in PyNs of *Scn2a^+/−^* mice impairs axonal and dendritic excitability, leading to synaptic dysfunction and deficits in learning and social behaviors [55]. Collectively, these findings highlight the intricate interplay between Na_V_ channel subtype localization, functional specialization, and the regulation of neuronal circuits, emphasizing their potential as precise therapeutic targets for neurological disorders. For instance, an United States Food and Drug Administration (US FDA)‐approved tetrodotoxin (TTX)‐sensitive Na_V_ channel blocker, riluzole, has shown clinical efficacy in patients with ALS [94]. Notably, the molecular structure of Na_V_ channels has informed the design of optogenetic tools for manipulating neuronal excitability, such as the AnkG‐binding domain of the intracellular loop II–III of Na_V_1.2 (ChR2–YFP–Na_V_II–III) and the Na_V_1.6 (ChR2–GFP–Na_V_II–III) targeted to the AIS [95, 96, 97].

#### 2.2.2 Voltage‐Gated Potassium Channel (K_V_)

Various K_V_ channels, akin to Na_V_ channels, are prominently localized at the AIS across diverse neuronal types [98]. These K_V_ channels are integral to maintaining the resting membrane potential, modulating the AP generation and repolarization, and regulating the neuronal firing frequency [64]. Key K_V_ channel subtypes identified at the AIS include K_V_7.2/7.3, K_V_2.1/2.2, and K_V_1.1/1.2/1.4 subunit, K_Ca_2.3, and K_2P_4.1 [66, 67, 72, 73, 98, 99]. Their distributions at the AIS exhibit pronounced cell‐type‐specificity. For instance, K_V_1 channels are exclusively expressed in the distal AIS of specific neuronal types through their interaction with the scaffold protein postsynaptic density‐93 (PSD‐93), such as neocortical and hippocampal PyNs, and neurons of nucleus magnocellularis, indicating subcellular specificity in their targeting and functional roles [56, 64]. Both K_V_7.2/7.3 and Na_V_ channels possess similar AIS targeting motifs, and K_V_7 channels are recruited to the AIS by binding of their C‐terminus to the AnkG N‐terminal [37, 100, 101]. K_2P_4.1, a mechanosensitive potassium leak channel, is critical for the rapid repolarization of the membrane [102]. Its C‐terminus domain contains an AnkG‐binding motif structurally homologous to those in Na_V_1 and K_V_7.2/7.3 channels [73]. K_V_1 channels at the AIS are heterotetramers composed of pore‐forming α subunits (K_V_1.1, K_V_1.2, and K_V_1.4) and accessory β subunits such as K_V_β2 [103, 104]. Unlike Na_V_1, K_V_7, and K_2P_ channels, K_V_1 channels lack AnkG‐binding motifs. Studies indicate that their recruitment and stabilization at the AIS depend on the synergistic effects of SCRIB and PSD‐93. Specifically, K_V_1 channels interact with SCRIB via a PDZ‐binding motif, and SCRIB serves as a critical adaptor that links the K_V_1 channel complex and PSD‐93 to AnkG [105]. K_V_7.2/7.3 channels, which are found at high densities in the distal AIS, critically influence neuronal excitability by setting the resting membrane potential and restoring it following an AP [106, 107, 108]. For instance, a mutation in K_V_7.3 channels in DA neurons of the VTA markedly disrupts AP generation and repetitive firing [70]. Strikingly, pharmacological activation of K_V_7.2/7.3 in *ANK2* (encoding ankyrin B (AnkB))‐conditional knockout mice, which show ASD‐related behavioral abnormalities and juvenile seizure lethality, restores neuronal excitability and rescues juvenile seizure‐related mortality, highlighting their therapeutic potential in neurological disorders characterized by hyperexcitability [98].

#### 2.2.3 Voltage‐Gated Calcium Channel (Ca_V_)

Ca_V_ channels, encompassing Ca_V_2.1 (P/Q‐type), Ca_V_2.2 (N‐type), Ca_V_2.3 (R‐type), and Ca_V_3 (T‐type) channels, have been identified at the AIS of diverse neuronal types, such as the layer V cortical PyNs, the cerebellar Purkinje neurons, and the GABAergic interneurons in the dorsal cochlear nucleus (DCN) [75]. In excitatory hippocampal neurons, prolonged depolarization activates both L‐type and T‐type Ca_V_ channels, elevating intracellular calcium concentration and triggering distal relocation and shortening of the AIS [109]. In hippocampal granule cells, the stimulation of muscarinic M1 receptors at the AIS activates Ca_V_3.2 channels, triggering a localized increase in intracellular calcium. The calcium signal, in turn, induces a negative shift of the K_V_7 activation curve, reducing shunting conductance and ultimately augmenting AP generation [110]. In inhibitory neurons of the DCN, the Ca_V_3.2 channels are functionally coupled to the dopamine D3 receptor (D3R) at the AIS. Their activity is modulated by protein kinase C (PKC), which suppresses Ca_V_3.2 channel function upon activation [111]. This DA regulation hyperpolarizes Ca_V_3‐dependent activation at the AIS, thereby suppressing burst neuronal firing and reducing neuronal output [111]. These findings collectively demonstrate the dual role of AIS‐localized Ca_V_ channels as both voltage sensors and critical regulators of neuronal excitability. Their dynamic modulation of firing properties not only underpins physiological signal processing but also contributes to the pathogenesis of neurological and psychiatric disorders.

### 2.3 Other AIS‐Related Proteins

The structural and subcellular functional integrity of AIS is governed by a complex interplay between its cytoskeletal scaffold, ion channels, and a diverse array of associated proteins. Core cytoskeletal components include βIV‐spectrin and αII‐spectrin tetramers, which form a periodic lattice that anchors key membrane complexes, such as AnkG and Na_V_ channels, to the underlying actin cytoskeleton. Extracellular signaling molecules like NrCAM and NF‐186 reach the AIS at later developmental stages and are dependent on AnkG [38, 112], mediated by a conserved FIGQY motif within their cytoplasmic domains [113]. Notably, AIS is ensheathed by a specialized ECM, consisting of aggrecan, brevican, neurocan, versican, and tenascin‐R [112, 114, 115, 116, 117, 118, 119]. Hedstrom et al. [112]. demonstrated that NF‐186 is required for recruiting brevican and versican to the AIS. Although the functions of the ECM at the AIS remain unclear, they may include the recruitment and clustering of AIS proteins, synaptic stabilization, and ionic buffering [120, 121]. Actin filaments, in addition to MTs, are integral components of the inner AIS shaft [122, 123]. Super‐resolution microscopic analyses have revealed numerous sub‐membranous, circumferential actin rings that extend along the axon and are intricately associated with the AIS complex [26]. These actin rings are spaced approximately 190 nm apart, interconnected by spectrin tetramers, and organized in a cyclic arrangement [16, 17].

MAPs and motor proteins at the AIS collectively coordinate and maintain the accuracy of bidirectional axonal transport [6, 124]. Moreover, the bundled MTs in AIS provide structural tracks and crucial directional information to support axonal transport. MTs serve as the structural tracks for motor proteins, including kinesin and dynein, which mediate long‐range bidirectional transport along the axons [125]. Kinesin family motors drive anterograde axonal transport, whereas the dynein family facilitates retrograde axonal transport [126]. Myosin, on the other hand, primarily regulates localized delivery of cargoes [127, 128]. Ndel1, another key player, localizes at the AIS by directly interacting with AnkG and dynein, thereby regulating polarized cargo transport [129]. MAP2 is implicated in the regulation of vesicle distribution via fine‐tuning the motor activities of KIF1 and KIF5 along the axon [130].

**FIGURE 1:**
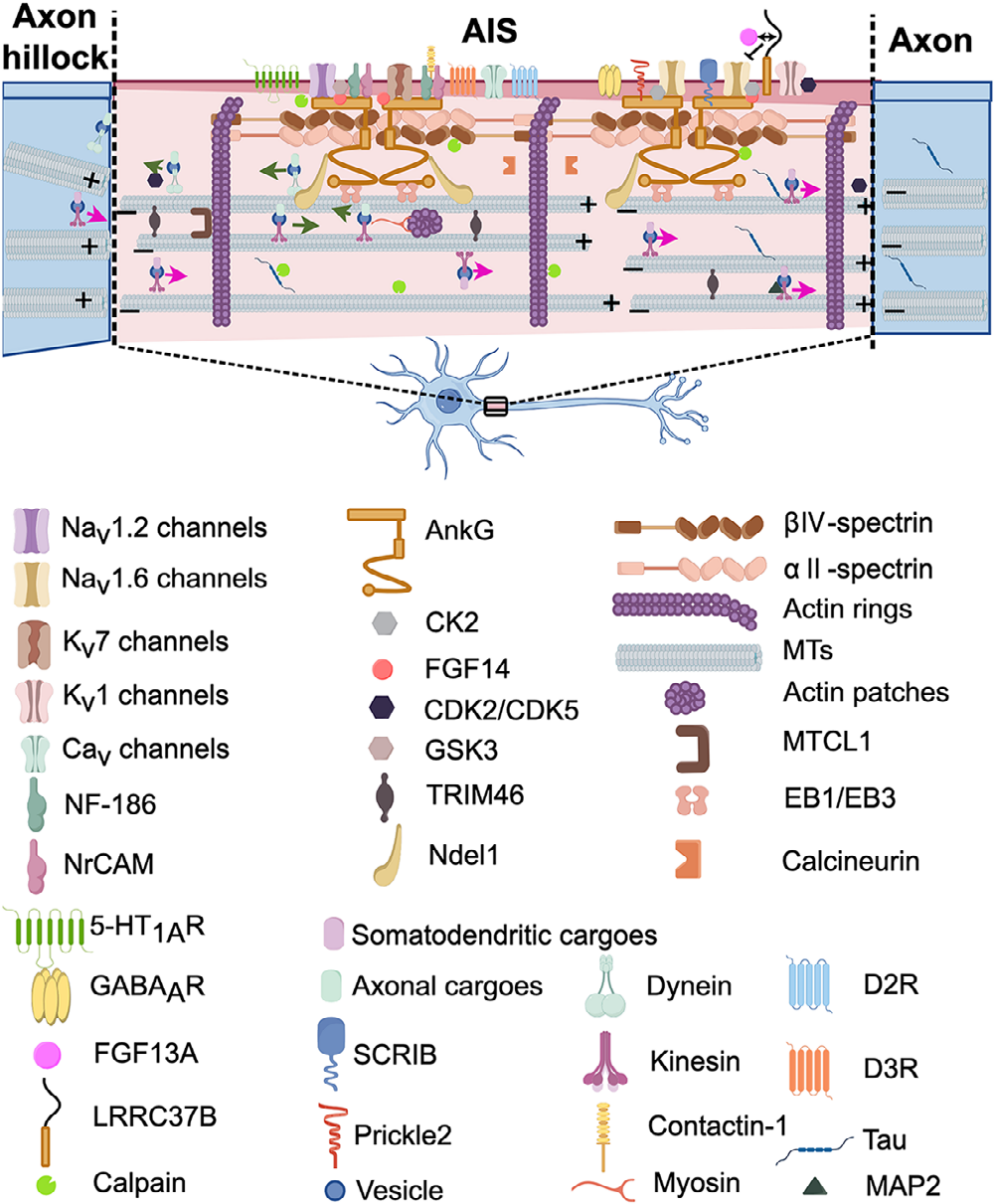
Molecular architecture of the AIS and its key protein components. AnkG serves as a scaffolding protein that recruits multiple partners and coordinates components across distinct AIS subdomains. The N‐terminal domain of AnkG anchors plasma membrane proteins, including voltage‐gated ion channels (critical for AP initiation), CAMs, and newly identified proteins. Various neurotransmitter receptors are also distributed across the AIS plasma membrane. A periodic submembrane network (∼190 nm spacing) consists of AnkG, βIV‐spectrin, and actin filaments, with spectrin linking actin rings to AnkG‐anchored membrane complexes. Within the inner shaft, AnkG associates with MT bundles, tau stabilizes MT bundles, and kinesin/dynein mediate cargo trafficking.

The molecular composition of AIS is further modulated by various protein kinases and proteases, including CK2, cyclin‐dependent kinases CDK2 and CDK5, calcineurin, GSK‐3, and calpain [21, 109, 131, 132, 133]. Recent studies have identified new targeted proteins, such as SCRIB, Prickle2, and Contactin‐1, which interact with AnkG, NF‐186, MT bundling, the Na_V_ channel, and LRRC37B, a receptor protein encoded by a hominid‐specific gene [119, 134, 135, 136]. However, the precise functional contributions of these proteins to AIS assembly and maintenance remain to be fully elucidated. Figure 1 provides a schematic representation of the major structural and functional components of the AIS discussed herein.

## 3 Mechanisms and Modulation of AIS Plasticity

The AIS exhibits remarkable plasticity, enabling neurons to tune their excitability and input/output in response to neuronal activity [109]. This section discusses the mechanisms and modulation of AIS plasticity, starting with its activity‐dependent changes, and subsequently elucidates the external signals that regulate the AIS plasticity.

### 3.1 Structural and Functional Plasticity

AIS plasticity is primarily manifested through changes in length, position, and molecular composition, which directly affect neuronal firing properties [137]. These activity‐dependent changes differ between excitatory and inhibitory neurons, enabling the neural network to adjust more flexibly [138, 139]. The following sections detail the activity‐dependent plasticity and its underlying molecular mechanisms.

#### 3.1.1 Activity‐Dependent Plasticity

Numerous studies have shown that AIS length and position can change with neuronal activity and cell‐type‐specificity of the brain region [137, 138]. These activity‐dependent changes, broadly defined as AIS plasticity, include alterations in AIS location and length, changes in ion channel expression, and even degradation of AIS proteins during excitotoxicity [9, 10]. These activity‐dependent changes further modulate AP propagation patterns, neuronal input/output properties, neuronal excitability, synaptic transmitter release, and initiation of disease pathogenesis [109, 137, 138, 140, 141, 142]. Usually, neurons with shorter AIS or closer to the cell body are less prone to excitation due to an increased AP firing threshold. Cultured hippocampal dentate granule cells (DGCs) with shortened AISs show decreased excitability due to a higher voltage threshold for AP firing [58, 109].

Structural plasticity of AIS can be classified into acute and long‐term forms based on its temporal dynamics following changes in neuronal activity [58, 109]. Evans et al. showed that activity‐dependent structural plasticity at the AIS can be induced much more rapidly. Just 3 h activity enhancement—by high K^+^ or patterned optogenetic stimulation—significantly shortens the AIS of hippocampal DGCs in a calcineurin‐dependent manner. This effect is associated with lower excitability and reduced repetitive spiking [109]. AIS shortening can also be rapidly induced within approximately 30 min by the transient activation of N‐methyl‐d‐aspartate (NMDA) receptors in excitatory hippocampal neurons (slices and culture) [58]. Therefore, the AIS can undergo rapid structural changes over timescales. These changes enable it to interact with other forms of activity‐dependent plasticity, thereby functioning in the dynamic control of neuronal excitability [58, 109]. Studies of avian brainstem auditory neurons reveal that auditory deprivation can increase AIS length in a few days, accompanied by increases in sodium current, membrane excitability, and spontaneous firing. This demonstrates that AIS length is regulated by presynaptic activity, a mechanism that may help maintain auditory pathway function after hearing impairment [138]. In cultured hippocampal neurons, 2 days of elevated network activity—induced by high K^+^ or patterned optogenetic stimulation—leads to distal AIS lengthening and a concurrent reduction in intrinsic excitability [137]. A similar homeostatic adaptation of the AIS has also been observed in human iPSC‐derived neurons [143]. To further elucidate the bidirectional nature of AIS plasticity, Jamann et al. further confirmed in behaving mice that prolonged (≥15 days) whisker deprivation induces long‐term AIS elongation in S1BF layer II/III PyNs and increased excitability, whereas brief (1–3 h) environmental enrichment triggers rapid AIS shortening, elevated spike threshold, and reduced neuronal output. Overall, these results indicate that the AIS and neuronal input–output properties are temporally diverse, bidirectional, and activity‐dependent, supporting its role for homeostatic adaptation under physiological conditions in vivo [144].

Compared with excitatory neurons, the AIS of inhibitory neurons exhibits distinct structural plasticity in response to changes in incoming activity levels. As observed in some typical inhibitory neurons, in dissociated cultures of the rodent olfactory bulb (OB), a subset of bulbar DA neurons exhibits different AIS plasticity [141]. Under baseline conditions, the AIS of these neurons is notably shorter and distally positioned along the axon. However, after chronic 24 h depolarization, it becomes longer and relocates proximally toward the soma, which is opposite to the direction change of the AIS plasticity in excitatory neurons [137, 138, 141, 145, 146]. The different forms of AIS plasticity in inhibitory and excitatory neurons may enable the neuronal network to adapt more flexibly to disturbances, thereby helping to maintain their stability and function [139].

#### 3.1.2 Molecular Mechanisms of Plasticity

Several proteins that contribute to AIS plasticity have been identified, including L‐type voltage‐gated calcium channels, calcineurin, myosin II/phospho‐myosin light chain, and the AKT pathway [109, 127, 137, 146, 147]. These findings are consistent with the observed remodeling of the AIS in disease or injury models [50, 148].

The activity of myosin II is regulated by phosphorylated myosin light chain (pMLC), and this process plays a crucial role in activity‐dependent changes in the organization of the AIS. pMLC is a component of AIS, and it is associated with actin rings. Its recruitment and/or stability depend on AnkG, and in turn, AIS assembly requires MLC phosphorylation and myosin II contractile activity. Due to the elevated pMLC levels, via aberrant activation of myosin II, it is sufficient to cause ectopic localization of AIS components, including AnkG, Na_V_, βIV spectrin, and NF‐186, to the distal axon. During depolarization, pMLC rapidly lost, thereby enabling activity‐dependent structural plasticity of the AIS via actin‐myosin II cytoskeletal scaffold destabilization [127]. Similarly, the Ca^2^^+^‐activated phosphatase calcineurin and L‐type Ca_V_1 calcium channels are required for activity‐dependent AIS structural plasticity after chronic or rapid activation in DGC of dissociated hippocampal cultures of rats or mice [109, 146]. Furthermore, Evans et al. indicate that CDK5 signaling maintains AIS length after elevated neuronal activity, partially by inhibiting calcineurin activity [109]. In contrast, within OB DA neurons, L‐type Ca_V_1 channels—but not calcineurin—mediate this plasticity, and the CDK5 maintains AIS structure by operating in a reverse manner upon depolarization through the same pathway [141]. Given the cell‐type‐specific nature of AIS plasticity, the precise interactions and relative contributions of these mechanisms across different cell types remain largely unresolved.

### 3.2 Regulators of the AIS Plasticity

Beyond the intrinsic activity patterns, AIS plasticity is also influenced by neurotransmitters and neuromodulators [58]. This section discusses how major signaling systems, such as glutamatergic and serotonergic transmission, regulate the AIS plasticity.

#### 3.2.1 Neurotransmitter and Neuromodulator Signaling

Emerging evidence indicates that extrasynaptic excitatory inputs can regulate AIS plasticity in glutamatergic neurons, despite the absence of glutamatergic synapses at the AIS. For instance, applying NMDA to acute tissue slices to activate NMDA receptors (NMDAR) induces significant structural reorganization of the AIS in CA1 PyNs. This reorganization is manifested by a reduction in AIS length, an increase in the distance between the AIS initiation point and the soma, and the absence of Na_V_1.2 channels in the distal AIS, ultimately leading to long‐term depression (LTD) [58]. Given the relatively sparse distribution of NMDARs at the AIS itself, this plasticity is likely mediated by the synaptic NMDARs located at somatodendritic sites, which rapidly propagate signals to the AIS [58]. Furthermore, under excitotoxic conditions, NMDA receptor subtype 2B (NR2B)‐containing NMDARs induce transient Ca^2+^ influx and subsequent calpain activation. This cascade promotes the rapid and irreversible endocytosis of AnkG‐associated K_V_7.2/7.3 and Na_V_1.2 channels [133]. This excitotoxicity‐dependent endocytosis is selective for AnkG‐bound AIS proteins, thereby altering AP generation and preventing excessive glutamate release [133]. Therefore, glutamatergic activity indirectly regulates AIS plasticity through modulation of the dynamics of ion channels and associated proteins at the AIS, highlighting a critical link between synaptic signaling and AIS functional adaptation.

**FIGURE 2:**
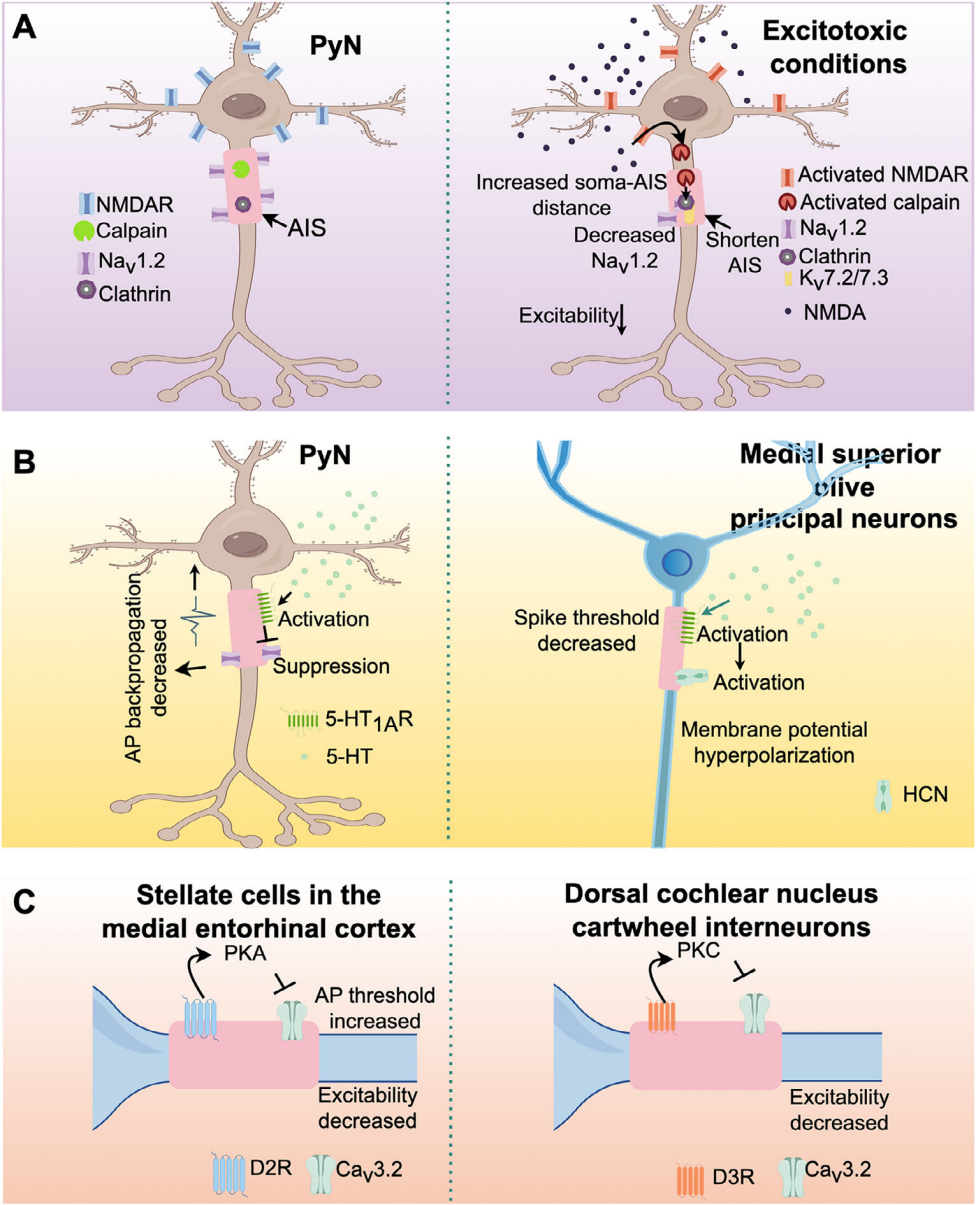
The AIS as a key site for neuromodulation. (A) Extra‐synaptic signals, mediated by somatodendritic NMDARs, can modulate AIS structure plasticity and cause loss of Na_V_1.2 channels, leading to LTD and preventing excessive glutamate release. (B) AIS‐targeted 5‐HT_1A_R modulation of neuronal excitability. (C) Dopamine receptors modulate neuronal firing by targeting T‐type Ca_V_3.2 channels at the AIS across diverse neuron types.

Similar to glutamatergic synapses, serotonergic boutons are absent from the AIS. However, certain 5‐HT receptors outside the AIS can regulate its morphology. In primary hippocampal neurons, overexpression of 5‐HT6R, which is localized at the primary cilia, increases the proportion of neurons with branched AIS, induces AnkG translocation to primary cilia, shortens the distance between the AIS and the soma, and increases the AIS length. Conversely, 5‐HT6R knockdown shortens the AIS [149, 150]. Consistent with these findings, primary hippocampal neurons of 5‐HT6R knockout mice exhibit shorter AIS lengths and a longer distance between the AIS and the soma, accompanied by elevated neuronal excitability compared with controls [151]. Thus, 5‐HT may affect neuronal excitability and polarity by regulating the AIS structure and ion activities of postsynaptic neurons in the neural circuit. This may have potential implications for the pathophysiology of neurological and psychiatric disorders, such as schizophrenia and depression (Figure 2).

## 4 Biological Functions

AIS has two principal functions: to initiate APs through its high‐density ion channels [77] and to control neuronal polarity by regulating the differential distribution and transport of proteins, organelles, and lipids between axonal and somatodendritic compartments [5, 6, 152]. The following sections detail the biological functions of AIS and the molecular mechanisms underlying it.

### 4.1 AP Initiation: Integration of Synaptic Inputs

Integrating synaptic inputs and generating APs is the core function of neurons. These APs serve as the fundamental electrical signals for neuronal communication and are crucial for synaptic signal transmission, sensory integration, and motor execution [153]. In most neurons, AIS serves as the initial and primary trigger site for the conversion from synaptic input to AP output [3, 153, 154]. After APs are initiated at the AIS, they propagate down the axon [155]. Additionally, the AP initiation at the AIS is directly modulated by the density of voltage‐gated ion channels expressed on the AIS [64, 77, 111].

In prefrontal PyNs, activation of 5‐HT_1A_R inhibits AP backpropagation by specifically suppressing the Na_V_1.2 subtype at the AIS, while concurrently enhancing axodendritic segregation [156]. Similarly, in principal neurons of the medial superior olive, 5‐HT_1A_R activation hyperpolarizes the membrane potential and lowers spike thresholds by regulating hyperpolarization‐activated cyclic nucleotide‐gated (HCN) channels [76]. In various neuronal types, dopamine receptors regulate AP generation by modulating T‐type Ca_V_ channels at the AIS. Specifically, in medial entorhinal cortex stellate cells, D2 receptors (D2Rs) activation at the AIS regulates the Ca_V_3.2 subtype through a protein kinase A‐dependent signaling pathway, thereby modulating AP thresholds [157]; in layer V prefrontal cortical PyNs of mice, D3Rs activation suppress AP generation by inhibiting Ca_V_3.2 channels at the AIS [158]; and in DCN cartwheel interneurons, D3R attenuates T‐type Ca_V_ channel activity via PKC signaling, thereby reducing AP output [159] (Figure 2). These parallel mechanisms demonstrate a conserved strategy in which dopamine signaling precisely controls neuronal output via Ca_V_ channels located at the AIS. However, the relationship between AIS structure and AP properties of DA neurons remains to be further studied. For instance, Meza et al. showed that in the SN, the size and position of the AIS correlate with the spontaneous firing rate of these neurons [160]. However, Moubarak et al. demonstrated that the resilience to AIS variation presented in DA neurons in the SN is primarily attributable to the complexity and excitability of the somatodendritic compartment. Their findings suggest that variations in AP properties and pacemaking frequency among DA neurons are not directly linked to AIS size or position [161]. These contrasting observations underscore the need for further investigation to clarify the precise relationship between AIS morphology and the functional properties of DA neuron activity in the SN.

### 4.2 Maintenance of Neuronal Polarization

Another critical function of the AIS is to maintain neuronal polarity [162]. The AIS maintains neuronal polarity through two principal mechanisms: (1) forming a diffusion barrier for membrane components at the cell surface [5] and (2) restricting the intracellular movement of vesicles and cytoplasmic components [6]. For instance, in cultured hippocampal neurons, 2 days after axon/dendrite differentiation, small molecules such as 10 kDa dextran, GFP, and DsRed can diffuse into the axon, whereas large molecules including 70 kDa dextran and BSA are excluded. These results indicate the existence of a selective filter at the AIS. This filter also selectively impedes the active transport of MT‐based vesicular carriers through cooperation with KIFs. It allows axonal entry of VAMP2, while restricting the entry of NR2B or GluR2 [6]. TDB has also been identified at the AIS, which facilitates the anterograde sorting of tau and prevents its retrograde return to the soma and dendrites [163]. This TDB is disrupted when tau is phosphorylated within its repeat domain and detaches from MTs [163], linking tau missorting and hyperphosphorylation with AIS filter dysfunction in neurodegenerative diseases.

Additional mechanisms regulate neuronal polarization and axonal transport. The kinesin complex KIF3/KAP3 plays a critical role in AIS assembly and neuronal polarization. In the developing axon with low MARK2 activity, nonphosphorylated KAP3 binds to and transports TRIM46 along MTs for accumulation at the nascent AIS. TRIM46 accumulation at the AIS subsequently stabilizes plus‐end‐out MT orientation, facilitating axon specification [124]. Moreover, in dendrites with high MARK2 activity, phosphorylation of KAP3 at S60 reduces its affinity with TRIM46, preventing mistargeting and ensuring proper neuronal polarity.

**FIGURE 3:**
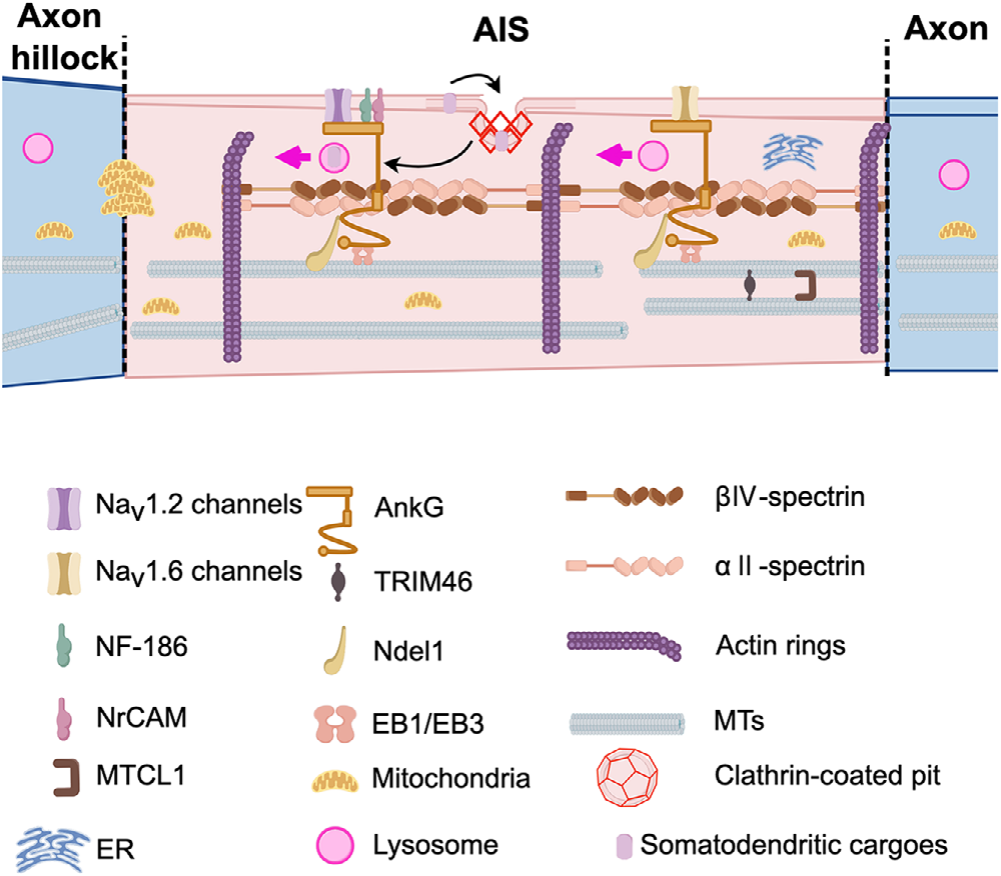
Endocytosis and organelle homeostasis at the AIS. Clathrin‐mediated endocytosis facilitates AIS protein internalization within the AIS, followed by potential lysosomal degradation, while mitochondrial clusters localize to the proximal AIS region.

Meanwhile, clathrin‐mediated endocytosis serves as another critical regulatory mechanism at the AIS, orchestrating the precise sorting of membrane proteins to stabilize, enrich, or eliminate key AIS components, such as Na_V_1.2 and K_V_7.2/7.3 channels. This process is indispensable for maintaining AIS homeostasis and facilitating activity‐dependent structural plasticity [23, 29, 58, 133, 164]. This endocytic process works synergistically with the AIS diffusion barrier to coordinate intracellular receptor sorting and plasma membrane dynamics, thereby preserving neuronal polarity. A hallmark of this system is its capacity to selectively remove polarized transmembrane proteins—including dendrite‐morphogenesis‐abnormal 1, transferrin receptor, and the serotonin G protein‐coupled receptor 1 (SER‐1) [165]—from the axonal membrane for subsequent degradation, thereby reinforcing axodendritic compartmentalization. Remarkably, this mechanism exhibits evolutionary conservation from *Caenorhabditis elegans* to humans, where it effectively eliminates polarity‐disrupting receptors, such as SER‐1 and the α‐amino‐3‐hydroxy‐5‐methyl‐4‐isoxazolepropionic acid receptor subunit GluA1 from the AIS [165]. Synaptic NMDAR activation triggers a rapid, clathrin‐dependent internalization of AIS Na_V_ channels within minutes, inducing LTD and elevating the AP threshold at the distal AIS [58]. Consequently, pathological overactivation of NMDA receptors may promote excitotoxic damage by disrupting AIS functional output and structural integrity through excessive endocytic removal of essential ion channels. This phenomenon provides a plausible mechanistic underpinning of excitotoxicity in various neurological disorders. Beyond its role in protein clearance, AIS‐associated endocytosis is vital for sustaining neuronal polarity and circuit functionality. Following internalization, voltage‐gated ion channels and other AIS proteins undergo divergent trafficking fates, including lysosomal degradation, ubiquitin–proteasome‐mediated processing, or localized recycling, collectively enabling comprehensive AIS remodeling [58, 165, 166] (Figure 3). In summary, the integrity of AIS and neuronal polarity are orchestrated by multiple interdependent mechanisms—including diffusion barrier, selective filtering, motor protein‐mediated transport, and endocytic regulation. These insights reveal the complex regulatory system operating at the AIS, both in physiological neuronal function and in the pathogenesis of neurological diseases.

### 4.3 Organelle Gatekeeping: Lysosomes and Mitochondria

Beyond its established roles in AP initiation and neuronal polarity, the AIS serves as a critical regulatory checkpoint for organelle trafficking and maintenance. Emerging evidence positions the AIS as a selective barrier that governs the axonal composition of organelles, including mitochondria, endosomes, and endoplasmic reticulum (ER)‐derived vesicles [166, 167]. This gatekeeping function suggests that the AIS may represent a vulnerable site where organelle dysfunction first manifests during neurodegenerative processes [168]. Notably, interventions targeting AIS‐specific mechanisms of organelle surveillance may offer novel therapeutic strategies. By preserving organelle integrity at this crucial bottleneck, we may potentially maintain neuronal homeostasis and mitigate the progression of various neurological disorders characterized by axonal degeneration. This paradigm shift in understanding AIS function highlights its dual role as both a master physiological regulator and a critical pathological sensor in maintaining organelle homeostasis.

#### 4.3.1 AIS as “Gatekeeper” for Lysosomal Traffic

Emerging studies now position the AIS as a functionally critical hub for coordinating organelle homeostasis, particularly lysosomal trafficking. Genetic studies have linked TMEM106B, a gene encoding a lysosomal transmembrane protein, to multiple age‐related neurodegenerative disorders, including limbic‐predominant age‐related transactive response DNA binding protein of 43 kDa (TDP‐43) encephalopathy, frontotemporal lobar degeneration, and PD [169, 170]. In TMEM106B knockout mice, motor neurons exhibit defective retrograde lysosomal transport and pathological accumulation of lysosome‐associated membrane protein 1‐positive vacuoles at the distal AIS. These disruptions are accompanied by lipofuscin and autophagosome aggregation, ultimately resulting in proximal axonal swellings and functional impairment of facial‐nerve‐innervated musculature—findings that substantiate the AIS's role as an organelle gatekeeper [167]. Complementing these findings, Purkinje cells in TMEM106B knockout mice exhibit analogous AIS‐specific pathologies, featuring vacuolated lysosomes and disrupted lysosomal trafficking, which correlate with measurable behavioral deficits, including motor dysfunction, gait abnormalities, and reduced startle responses [168, 171]. The presence of ER components in the AIS suggests additional regulatory complexity, as lysosomal disturbances at this site may involve ER–lysosome membrane contact sites, thereby disrupting ER‐phagy and leading to sequential neuronal dysfunction [172, 173].

Collectively, these findings underscore the integral role of the AIS in maintaining proper retrograde axonal transport and lysosomal function. The AIS emerges not merely as an electrophysiological trigger zone, but as a critical organizer of neuronal organelle homeostasis whose dysfunction may represent an early event in neurodegenerative pathogenesis.

#### 4.3.2 AIS in Association With Mitochondrial Axonal Transport

The AIS exerts precise control over mitochondrial trafficking, governing their distribution between the soma and distal axonal compartments. Following nerve injury, proteasome‐mediated transient disassembly of the AIS facilitates mitochondrial redistribution into damaged axons, where they provide essential metabolic support for regeneration and structural maintenance [166]. Notably, mitochondrial distribution within the AIS exhibits spatial heterogeneity: mitochondria clusters exist in the proximal AIS, while remaining sparse in the central segments. These mitochondria clusters in the proximal AIS exhibit limited mobility, with only a small fraction undergoing active axonal transport [174]. It has been reported that these mitochondria clusters contribute to AD pathogenesis, as their local disruption leads to significant accumulation of somatic tau [174], in relation to tauopathy. Moreover, amyloid‐β (Aβ) oligomers disperse mitochondrial clusters from the proximal region toward central segments, though the mechanistic relationship between AD pathology formation and AIS‐mitochondria translocation remains unknown [175]. Thus, the physiological significance of AIS‐resident mitochondria remains to be fully defined. Further studies are needed to unravel the role of AIS‐located mitochondria in regulating AIS function and neuronal polarity, with special focus on their pathological effect in tempering tau anterograde sorting and AD pathogenesis. Figure 3 illustrates the compartmentalized organization of AIS subdomains under physiological conditions.

### 4.4 Regulation of Neural Circuit Excitability and Homeostasis

The function of AIS is also dynamically regulated by diverse extrinsic signals. These extrinsic signals include specialized axo‐axonic inhibitory innervation, regulation by neurotransmitters such as serotonin, and dynamic interactions with glial cells [35, 176, 177]. These mechanisms collectively indicate that AIS is a key domain for regulating neuronal excitability and circuit dynamics.

#### 4.4.1 Axo‐Axonic Innervation

AIS receives innervation from GABAergic axo‐axonic interneurons, which are essential components of neural circuits [178]. In the mammalian cerebral cortex, hippocampus, and basolateral amygdala, the AIS of PyNs receive specialized inhibitory inputs from chandelier cells (ChCs), which are also known as axo‐axonic cells (AACs) [35, 179, 180, 181]. These ChCs specifically target the AIS of local glutamatergic PyNs, while avoiding other subcellular domains and GABAergic interneurons [182]. Recent work revealed that AACs are distributed throughout almost all the pallium‐derived brain structures [183]. Each AACs innervate hundreds of nearby PyNs, while a single AIS is often innervated by multiple AACs [184, 185]. Therefore, these interneurons are believed to play a crucial role in regulating AP generation and neuronal excitability, thereby regulating network operations and information processing [183]. These functions play a critical role in orchestrating higher‐order cognitive processes such as working memory [186, 187]. For instance, during sharp waves—which are essential for memory consolidation—the firing of CA3 AACs is suppressed in vivo [188]. This suppression thereby reduces GABA release at the AISs of CA3 PyNs temporally and spatially [189], which is required for their subsequent reactivation [190]. Similarly, during theta oscillations, CA3 ChCs are inhibited by specific GABAergic neurons originating from the medial septum [189]. This suppression of ChCs leads to PyN disinhibition, thereby promoting the generation of the theta dipole. Collectively, these findings indicate that during both sharp wave–ripple and theta oscillation, CA3 ChCs act as a key regulator of CA3 PyNs activity. In another study, ChCs mediate learning‐dependent refinement by selectively inhibiting individual PyNs rather than globally. Following motor learning, ChCs exert differentially strengthened control over specific PyN subsets, with significantly different input intensity to the AIS across cells [185]. This diversity reflects the capacity of ChCs to adaptively regulate inhibitory strength based on the target cell properties, thereby shaping PyN functional properties. Consistent with this, blocking ChC synaptic transmission in the motor cortex perturbs the accuracy of directional movement control, indicating their essential role in fine‐tuning cortical circuits during motor learning.

It is important to note that while ChCs provide a prominent source of inhibition at the AIS, a substantial percentage of GABAergic synapses at the AIS are from non‐ChCs [185]. For example, in the cerebellum, basket and stellar interneurons project specific axon terminals to the AISs of Purkinje cells, forming GABAergic “pinceau” synapses [191]. Unlike the extensive synaptic cartridges of ChC‐derived AIS connections, most non‐ChC‐derived AIS connections contain only one synapse. The formation and maintenance of GABAergic synapses at the AIS depend on a complex interplay of molecular components, such as γ‐aminobutyric acid type A receptors (GABA_A_Rs) [179], AnkG [35], βIV‐spectrin [192], L1CAM [193, 194], neurofascin [180, 195], Erbb4/Dock7 [196], L1CAM [193], fibroblast growth factor13 [197], IgSF11 [198], and Cntn‐1 [119]. At the AIS, GABA_A_R α2‐subunits interact with collybistin, a key protein that interacts with gephyrin, an inhibitory receptor anchoring protein. Hines et al. generated Gabra2‐1 mice by substituting amino acids within the GABA_A_R α1 loop into the GABA_A_R α2‐subunit region responsible for collybistin binding. Gabra2‐1 mice show decreased GABA_A_R cluster size, leading to inhibitory synaptic transmission, heightened anxiety, developmental seizures, and premature mortality, suggesting that GABA_A_R at the AIS plays a key role in brain activity [199]. AnkG stabilizes GABAergic synapses at the AIS and somatodendritic sites of excitatory PyNs by directly interacting with the GABARAP [31]. In the AnkG W1989R mutant mouse model, the mutation abolishes the AnkG/GABARAP interaction and reduces GABAergic synaptic connectivity in layer II/III of the somatosensory cortex and the hippocampal CA1 region. This reduction in inhibitory synapses causes hyperexcitability of PyNs in the cortico‐hippocampal circuits, accompanied by AIS shortening, diminished excitatory postsynaptic currents, decreased dendritic spine density, and defective gamma oscillations, indicative of network desynchronization [200].

These studies highlight the critical role of axo‐axonic innervation in regulating neuronal excitability and network synchronization. Deficiencies in these molecular components at the AIS impair the density of GABAergic synapses on the AIS, leading to impaired neuronal excitability, disrupted synaptic plasticity [180], neurodevelopmental abnormalities, and psychiatric disorders, including schizophrenia [201], BD [200], and AD [202]. These studies highlight the axo‐axonic innervation as a critical neural circuit for understanding the pathophysiology of these neuropsychiatric and neurodegenerative diseases.

#### 4.4.2 Serotonin

The AIS and the 5‐HT_1A_Rs expressed on it constitute a critical component in the 5‐HT‐mediated regulation of motor neuron circuits [176]. Research reveals that 5‐HT exerts a bidirectional regulatory effect on the activity of motor neurons: during low‐intensity activity, 5‐HT enhances neuronal excitability primarily through 5‐HT_2_Rs located in somatodendritic regions. In contrast, during sustained or high‐intensity activity, although no serotonergic synapses are present at the AIS, 5‐HT spills over and diffuses to the AIS activating extra‐synaptic inhibitory 5‐HT_1A_Rs [203]. The mechanism has been confirmed in humans through a placebo‐controlled study demonstrating that Buspirone, a 5‐HT_1A_R partial agonist, significantly reduces motoneuron excitability [204]. The study concluded that the inhibitory effect of Buspirone may occur through the activation of 5‐HT_1A_R at the AIS, providing direct evidence for this pathway in human motor control. These AIS‐localized 5‐HT_1A_Rs inhibit AP generation, thereby preventing motor neurons from becoming hyperexcitable and avoiding the occurrence of spastic muscle contractions [203]. This mechanism resolves the seemingly contradictory phenomenon exhibited by 5‐HT, which facilitates muscle contraction and induces central fatigue. It also indicates the AIS as a key nonsynaptic integration site within 5‐HT circuits, essential for preventing neuronal hyperexcitability and maintaining motor system homeostasis (Figure 2).

#### 4.4.3 Glial Modulation of AIS Structure and Function

Emerging evidence demonstrates that glial cells actively regulate neuronal excitability through dynamic interactions with the AIS. Microglia maintain continuous contact with the AIS from early development through adulthood, exerting tonic modulatory effects on neuronal activity. In the adult mouse cortex, a specialized subset of perineuronal satellite microglia is preferentially located on the axonal side of the soma, with 47% of these cells extending processes that closely associate with over half the AIS length [177]. These specific AIS‐associated microglia emerge early in development, showing increased frequency and more prominent localization at the proximal AIS from P9 to P30. This period that follows the established formation of cortical AISs [177]. Functionally, these neuron–microglia interactions significantly impact neuronal excitability. This is confirmed in coculture experiments using human iPSC‐derived models. In the experiment, microglia are cocultured with Na_V_1.2–L1342P neurons carrying a seizure‐related mutation. The presence of microglia substantially reduces both sodium current density and repetitive firing in the hyperexcitable Na_V_1.2–L1342P neurons. This occurs along with a decreased expression of sodium channels at the AIS region of neurons [205]. Notably, disruption of microglial‐AIS interactions following traumatic brain injury (TBI) correlates with aberrant neuronal firing patterns [177]. Microglial phenotypic states differentially influence AIS integrity, proinflammatory microglia promote AnkG disassembly and AIS destabilization, while anti‐inflammatory microglia facilitate AIS structural recovery [206]. Under physiological conditions, microglia support the formation of axo‐axonic synapses between PyN AIS and ChCs, a process that is impaired during microglial activation [207]. These findings collectively indicate that microglia are active regulators of neuronal excitability, exerting their effects through structural and molecular modifications at the AIS. This microglia–AIS interaction is thus critical for maintaining physiological homeostasis, and its dysregulation may contribute to disease pathogenesis, underscoring the glial‐neuronal essential role in normal brain function.

**FIGURE 4:**
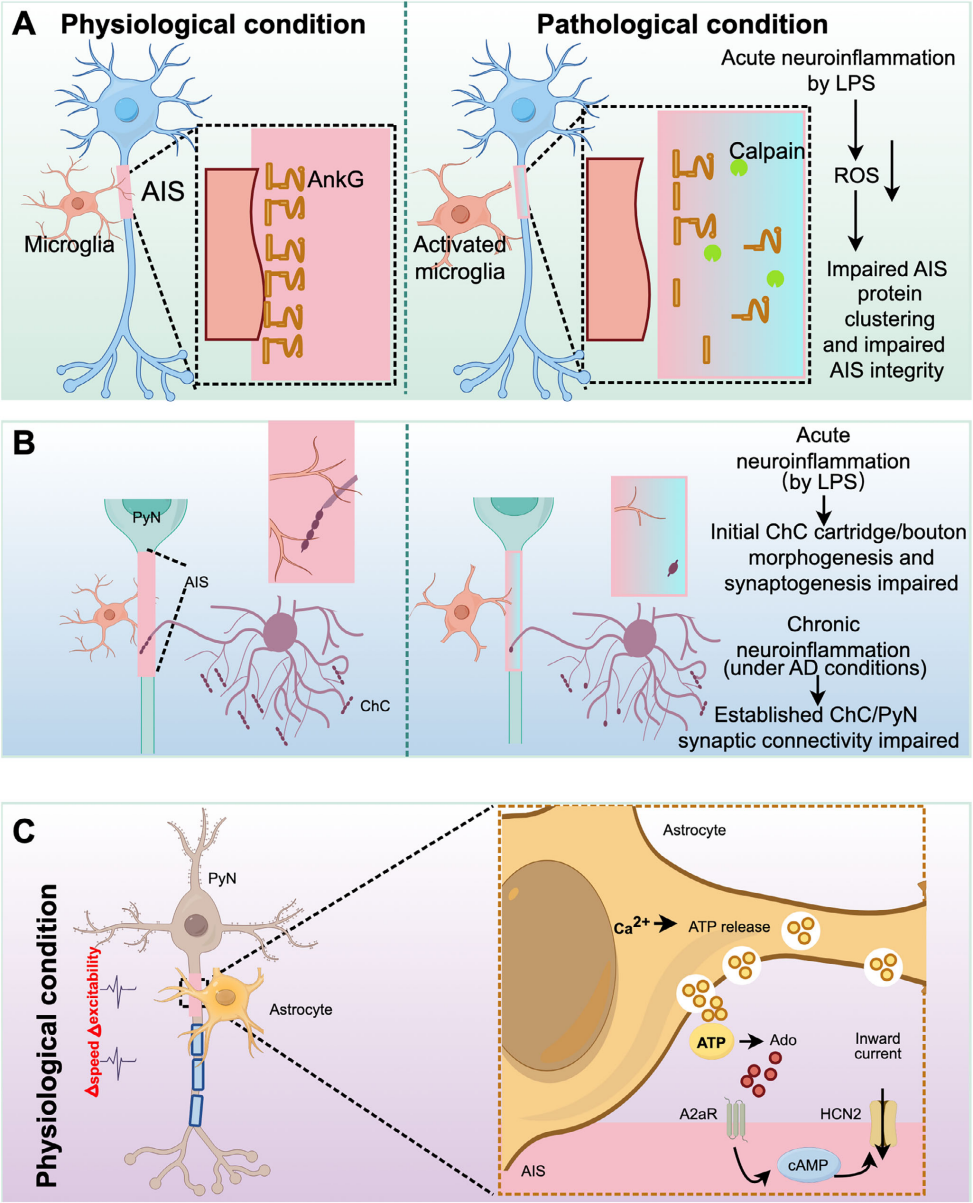
Glial regulation of AIS in health and disease. (A) Microglia maintain AIS integrity under physiological conditions but promote AnkG disassembly via calpain‐mediated proteolysis during neuroinflammation. (B) In the healthy brain, microglia facilitate synaptogenesis between PyN AISs and ChCs. Microglial dysfunction or aberrant activation disrupts this precise synaptic organization. (C) Astrocytes indirectly regulate AIS function through calcium‐dependent vesicular ATP release. Following extracellular conversion to adenosine, activation of A_2a_ receptors at the AIS and nodes of Ranvier elevates cAMP levels. This enhances HCN channel activity, generating inward currents that modulate AP initiation, conduction velocity, and overall neuronal excitability (modified from [208]). LPS, lipopolysaccharide; ROS, reactive oxygen species.

Astrocytes similarly modulate AIS function through multiple mechanisms. Calcium‐dependent vesicular ATP release from astrocytes undergoes extracellular conversion to adenosine, which subsequently activates Gs‐coupled A_2a_ receptors at the AIS and nodes of Ranvier. This signaling cascade elevates intracellular cAMP levels and activates HCN channels, ultimately modifying AP initiation, axonal conduction velocity, and overall neuronal excitability [208]. Astrocyte‐encoded Semaphorin3a additionally governs AIS spatial orientation in α‐motor neuron [209]. Moreover, astrocytes in postnatal APP/PS1 mice exacerbate AIS structural damage by impairing retinoic acid synthesis, thereby precipitating an early imbalance between excitation and inhibition [210]. These findings collectively indicate that astrocytes actively regulate AIS function through diverse mechanisms, including dynamic modulation of ion channels and structural maintenance. These mechanisms finely modulate neuronal excitability under physiological conditions and lead to dysfunction under pathological conditions (Figure 4).

## 5 Disease Spectrum

Impaired structural and functional plasticity of the AIS contributes to the pathogenesis of multiple nervous system diseases, including AD, FTD, ALS, epilepsy, schizophrenia, BD, TBI, and pain [8, 12, 50, 57, 211, 212, 213, 214, 215, 216].

### 5.1 Neurodegenerative Diseases

#### 5.1.1 Alzheimer's Disease

AD and related tauopathies, including FTD with tau inclusions, are characterized by dysregulated neuronal excitability. The AIS is a key regulator of neuronal excitability and has been implicated in the pathological neuronal hyperactivity observed in AD. Studies have documented both impaired structural plasticity and defective function of AIS in both AD patients and mouse models [11, 211]. In postmortem hippocampal tissues from individuals with AD, both the mean concentration of TRIM46 within the AIS and the AIS length itself are statistically significantly reduced in neurons with “tau accumulation without NFTs” or with “NFTs present” compared with neurons with “no tau accumulation” [217]. Consistent with these human findings, 6‐month‐old APP/PS1 mice exhibit decreased AnkG expression and AIS shortening in hippocampal CA1 neurons, leading to impaired AIS plasticity and slower AP propagation [8]. In primary mouse cortical neuron cultures, extracellular tau oligomers selectively reduce AIS length from the distal end and diminish TRIM46 protein levels specifically within the AIS, without altering total cytoplasmic TRIM46 [217]. Hyperphosphorylated tau in hippocampal CA1 neurons of P301L tau transgenic mice induces distal AIS relocalization, suppressing hippocampal excitability [49], while acetylated tau renders MTs hyperdynamic and compromises the cytoskeletal sorting machinery within the AIS in primary rat hippocampal neurons caused by MTs [218]. Similarly, the FTD‐associated V337M tau mutation shortens the AIS and impairs its plasticity and the homeostatic control in human iPSC‐derived cortical neurons, likely through the abnormal submembrane accumulation of EB3 [50]. Additionally, intracellular tau‐ACs impair tau‐MT interactions, leading to impaired AIS plasticity and pathological tau mislocalization in primary hippocampal neurons [48]. Therefore, AIS may be one of the early and major targets of tau‐mediated pathogenesis in AD and related tauopathies.

**FIGURE 5:**
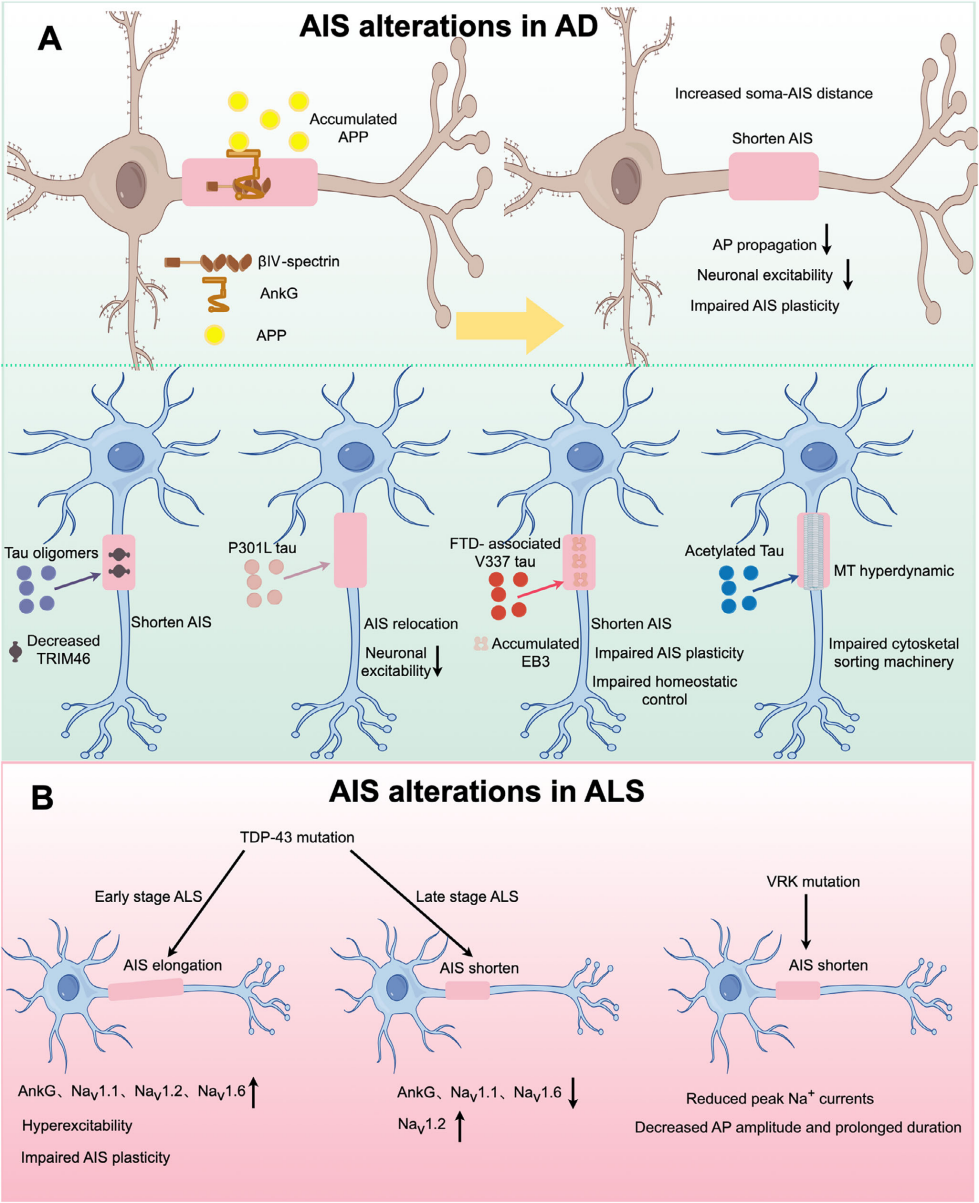
AIS alterations in various neurodegenerative disorders. (A) In AD, the AIS emerges as a key early target. Both tauopathy and APP/Aβ pathology disrupt its structure and function, thereby dysregulating neuronal excitability. (B) ALS‐linked mutations disrupt AIS structure and function, thereby impairing neuronal excitability.

While extracellular Aβ plaques constitute a defining neuropathological feature of AD, emerging evidence indicates that accumulating amyloid precursor protein (APP) shortens AIS independent of Aβ production in primary mouse cortical neurons overexpressed with mutant human APP [11]. APP forms physiological complexes with AnkG and βIV‐spectrin at the AIS, and its elevated expression, whether through glutamatergic stimulation, genetic overexpression, or pathogenic mutations, induces progressive AIS shortening and distal displacement from the soma. These structural alterations correlate with diminished neuronal excitability [11]. In mouse cortical neuron cultures, APP colocalizes with the Na_V_1.6 channel, and coimmunoprecipitation assays confirm their interaction in both mouse brain and HEK293 cells, though these assays do not distinguish between direct or indirect binding. Functionally, knockdown of endogenous APP in HEK293 cells stably expressing Na_V_1.6 significantly reduces sodium current density, indicating that APP expression enhances sodium currents in this mammalian cell system [219]. Knockdown of Na_V_1.6 channel in the hippocampus of APP/PS1 transgenic mice decreases the accumulation of Aβ by suppressing the cleavage of APP via reducing the transcription level of β‐site APP‐cleaving enzyme 1 [220]. In APP/PS1 transgenic mice, Na_V_1.6 suppression rescues cognitive deficits, normalizes synaptic function, and ameliorates abnormal hippocampal hyperexcitability [220]. These studies suggest links between Aβ pathology and the structure and function of AIS proteins, offering new insights into the early intervention in amyloid formation and neural hyperactivity (Figure 5).

#### 5.1.2 Amyotrophic Lateral Sclerosis

ALS is a neurodegenerative disorder characterized by selective motor neuron degeneration, in which dysregulated neuronal excitability emerges as a hallmark pathological feature. The disease progression exhibits a biphasic excitability profile, transitioning from early hyperexcitability to late‐stage hypoexcitability. AIS homeostatic plasticity plays a critical role in regulating neuronal intrinsic excitability.

Evidence from the TDP‐43(ΔNLS) mouse model demonstrates that cytoplasmic TDP‐43 aggregation—a pathological hallmark present in ∼97% of ALS cases—is sufficient to induce reversible AIS remodeling characterized by lengthening and constriction. This structural plasticity directly contributes to spinal motoneuron hyperexcitability by reducing rheobase, increasing gain, diminishing afterhyperpolarization, and enhancing persistent inward currents. Importantly, both the AIS alterations and associated excitability changes were reversible upon transgene suppression, indicating a dynamic form of homeostatic plasticity rather than irreversible damage [221].

The fact that similar excitability changes are observed in the human iPSC‐derived motor neurons with TDP‐43 mutation. At early disease stages, these neurons exhibit significant AIS elongation coupled with impaired activity‐dependent plasticity. These structural changes correlate with increased expression of AnkG and AIS‐specific voltage‐gated sodium channels Na_V_1.1, Na_V_1.2, and Na_V_1.6, resulting in pathological hyperexcitability. Conversely, late‐stage ALS motor neurons (both iPSC‐derived and postmortem specimens) exhibit AIS shortening accompanied by reduced AnkG, Na_V_1.1 and Na_V_1.6 expression, though Na_V_1.2 levels remain elevated, suggesting stage‐specific channelopathy mechanisms [12].

Further mechanistic insights come from studies on the mutations related to the vaccinia‐related kinase (VRK1) in patients with ALS. VRK1‐mutant human iPSC‐derived motor neurons display reduced peak sodium currents, AIS shortening, and consequent electrophysiological abnormalities including decreased AP amplitude and prolonged duration [222]. Collectively, therapeutic modulation of the AIS at the early disease stages is expected to become a potential strategy for counteracting the initial excitability imbalance in ALS (Figure 5).

### 5.2 Neuropsychiatric and Neurodevelopmental Disorders

#### 5.2.1 Epilepsy

Multiple epilepsy‐associated genes encode essential AIS components, including *SPTAN1* (encoding αII‐spectrin), *SCN1A* (encoding Na_V_1.1)*, SCN2A*, and K_V_ channels [59, 65, 69, 212, 223]. This genetic convergence firmly indicates the AIS as a critical node in epilepsy pathophysiology. *ANK2* deficiency exemplifies this mechanism, with loss‐of‐function variants causing hyperexcitable, desynchronized neuronal networks, aberrant somatodendritic arborization, and maladaptive AIS plasticity in human iPSC [13]. Mutations in *TBC1D24*, which encodes a multifunctional protein involved in synaptic vesicle trafficking, are associated with both mild and severe epilepsies, as well as complex syndromic phenotypes. In *TBC1D24*‐silenced rat primary cortical neurons, impairments in AIS maturation and AP firing have been reported [224]. Dominant pathogenic mutations in the *KCNT1* gene, encoding the Na^+^‐activated K^+^ channel Slack (K_Na_1.1), further highlight this mechanism. Studies in cultured cortical neurons and layer II/III of the mouse frontal cortex demonstrate that *Slack* mutations, such as the *R455H* variant, cause significant AIS elongation in both PyNs and GABAergic interneurons. This structural change is accompanied by upregulated expression of Na_V_1.2 and Na_V_1.6 at the AIS. The consistent AIS elongation observed across both excitatory and inhibitory neuronal populations may perturb excitation–inhibition balance within neural circuits, providing a structural basis for understanding network hyperexcitability in epilepsy [225]. The *OTUD7A L233F* variant, another epilepsy‐associated mutation, disrupts interactions with AnkG and AnkB. In mouse primary cortical neurons or human *15q13.3* microdeletion and *OTUD7A^L233F/L233F^* iPSCs, AnkG expression and stability are decreased, while its polyubiquitination is increased. These molecular changes are accompanied by impaired axonal growth and impaired intrinsic excitability. Importantly, these pathological phenotypes are reversible through AnkG restoration in *15q13.3* microdeletion neurons, suggesting a critical role of AnkG in neuronal development [226]. These findings collectively link AIS dysfunction to epilepsy pathogenesis, offering novel therapeutic targets for precision interventions (Table 1).

#### 5.2.2 Schizophrenia and BD

Schizophrenia manifests as a complex neuropsychiatric disorder characterized by psychosis, sensory processing deficits, and cognitive impairment. Recent evidence suggests that pathological alterations in ChC–PyN axo‐axonic synapses at the AIS may be a potential substrate for cortical circuit dysfunction in this disorder [214]. Postmortem analyses of patients with schizophrenia demonstrate elevated immunoreactivity levels for GABA_A_R α2 subunits at the AIS of principal cortical neurons [227]. GWAS further identifies *ANK3* as a significant schizophrenia risk locus [228]. Complementary findings reveal disease‐associated variants in voltage‐gated sodium channels (particularly *SCN2A*) and potassium channels (e.g., K_V_3.1), suggesting widespread ion channel dysregulation at the AIS [60, 68]. This evidence provides a niche for restoring cortical excitability and synaptic signaling in schizophrenia through targeting AIS dysfunction, such as GABA_A_R α2 subunit anomalies, *ANK3* variants, and ion channel dysregulation (Table 1).

BD, a debilitating psychiatric condition marked by alternating manic and depressive episodes [229], is similarly linked to genes encoding AIS localized proteins, where their roles in this compartment hold particular pathophysiological relevance [14, 61, 74]. GWAS and whole‐exome sequencing studies consistently identify *CACNA1B* (encoding Ca_V_2.2), *SCN2A*, *KCNB1* (encoding K_V_2.1) and *ANK3* as significant risk factor genes for BD [14, 61, 74]. Notably, *ANK3* emerged as one of the first genes demonstrating causative significance in BD pathogenesis [200, 230]. Mechanistic studies reveal that conditional knockout of *ANK3* exon 1b in mouse parvalbumin‐positive interneurons reduces sodium channel density at the AIS, elevates neuronal firing thresholds, and compresses AP dynamic range. These electrophysiological disturbances correlate with behavioral phenotypes mirroring BD symptomatology, including epilepsy susceptibility and sudden death in murine models [231]. Similarly, a significant decrease in DNA methylation in the *KCNQ3* (encoding K_V_7.3) exon 11 region was also observed in postmortem BD brains, indicating that *KCNQ3* variation may contribute to BD pathogenesis by causing ion channel dysfunction and eventually channelopathy [71]. Although these proteins are expressed beyond the AIS, their critical functions at the AIS position place this compartment as a critical node where genetic vulnerabilities converge to disrupt neuronal excitability and network function in BD (Table 1).

#### 5.2.3 Autism Spectrum Disorder

ASD is characterized by core behavioral phenotypes including social deficits, repetitive behaviors, and cognitive dysfunction. Emerging genetic evidence implicates de novo mutations affecting AIS components as significant contributors to ASD etiology. A key finding is the frequent implication of *SCN2A*, which encodes the Na_V_1.2 channel expressed at the AIS, making it one of the most commonly affected genes in ASD [232]. The loss of Na_V_1.2 in mouse prefrontal cortex mature PyNs impairs somatodendritic excitability, synaptic function, and AIS plasticity, ultimately impairing learning and social behavior [55]. Whole‐exome data of ASD patient cohorts identify pathogenic variants in *ANK3*, a gene critical for AIS organization and maintenance [233]. In deep sequencing of ASD patient brain tissues, *SCN1A*, *SCN2A*, *SCN8A*, and *CACNA1H* (encoding Ca_V_3.2) exhibit recurrent loss‐of‐function and/or deleterious missense mutations [15, 53]. Notably, K_V_7.3 mutations in midbrain DA neurons alter AP generation and firing patterns in murine models, providing a potential mechanistic link to ASD‐associated behavioral deficits [70]. Experimental models further demonstrate that selective deletion of *Tsc1* from cerebellar Purkinje neurons—known to cause ASD‐linked behavioral impairments—induces significant AIS alterations, including reduced AnkG immunoreactivity and decreased Na_V_ channel density, particularly affecting the fast‐transient Na_V_ current. These molecular changes result in a depolarized shift in the AP threshold, impaired AP initiation at the AIS, and a prolonged delay between AIS and somatic AP propagation, indicating compromised AP backpropagation into the somatic compartment [234]. Collectively, these findings indicate voltage‐gated ion channels at the AIS as critical nodes in ASD pathogenesis, where their dysfunction may disrupt neural circuit activity and contribute to disease‐relevant behavioral phenotypes (Table 1).

### 5.3 Other Neurological Conditions

#### 5.3.1 Traumatic Brain Injury

TBI is caused by external mechanical forces that disrupt both brain parenchyma and supporting structures, with traumatic axonal injury representing a primary neuropathological hallmark that may initiate downstream degenerative cascades. Intriguingly, experimental studies in murine mild TBI models reveal preferential localization of axonal damage to the AIS and the adjacent para‐AIS regions within the neocortical gray matter [216]. A concussion is also a type of TBI [235]. In Thy1–YFP‐H mice subjected to mild central fluid percussion injury, rapid structural disintegration of AnkG clusters occurs within 24–48 h postinjury. This is accompanied by dispersal of Na_V_1.6 channel aggregates, decreased neuronal excitability, axonal sprouting, and an expansion of the AIS (characterized as a wider, distended structure with diminished AnkG staining intensity) in axotomized layer V PyN sub‐population [235]. The temporal correlation between early AIS disruption and subsequent axonal pathology suggests that stabilizing the AIS represents a potential neuroprotective strategy to mitigate progressive tissue damage following TBI.

#### 5.3.2 Pain

The dorsal horn of the spinal cord harbors specialized microcircuits that process nociceptive information, comprising precisely interconnected excitatory and inhibitory neurons that converge onto projection neurons relaying signals to supraspinal structures. Existing evidences demonstrate that activity‐dependent plasticity of the AIS in these dorsal horn neurons critically regulates pain sensitivity. In inflammatory pain states, inhibitory interneurons exhibit a distal AIS repositioning, leading to decreased intrinsic excitability. This maladaptive plasticity disrupts normal inhibitory tone within dorsal horn networks, potentiating nociceptive transmission and promoting hyperalgesia [236]. Furthermore, in dorsal root ganglion neurons, the AIS located in proximal axonal segments generates ectopic discharges through clustered Na_V_1.7 channels, directly mediating mechanical allodynia in neuropathic pain conditions [57]. The demonstrated role of AIS plasticity in both inflammatory and neuropathic pain suggests that targeted modulation of AIS properties could represent a novel therapeutic strategy to minimize aberrant nociception and alleviate chronic pain.

## 6 Therapeutic Targets and Interventions

Since the impaired structural and functional plasticity of the AIS is a core pathology in various neurological diseases, it represents a potential therapeutic target. This section explores the potential and challenges of targeting AIS‐related proteins such as AnkG and various voltage‐gated ion channels, as well as the innovative approaches that can harness AIS plasticity for circuit repair.

### 6.1 AIS‐Associated Proteins as Targets

AnkG, as the central organizer of the AIS, is indispensable for its formation and the maintenance of neuronal polarity, underscoring its potential to be a primary therapeutic target [34]. Pathological studies have further confirmed the potential of AnkG. In diseases such as AD [8], TBI [235], and late‐stage ALS [12], the expression of AnkG decreases or its clusters disintegrate. Furthermore, *ANK3* is a significant risk locus for schizophrenia [228], BD [14, 230], and ASD [233]. The most compelling evidence for AnkG therapeutic validity comes from the *15q13.3* microdeletion model, in which the restoration of AnkG function can reverse pathological phenotypes, directly demonstrating that stabilizing the AIS scaffold through AnkG can rescue neuronal function [226].

**FIGURE 6:**
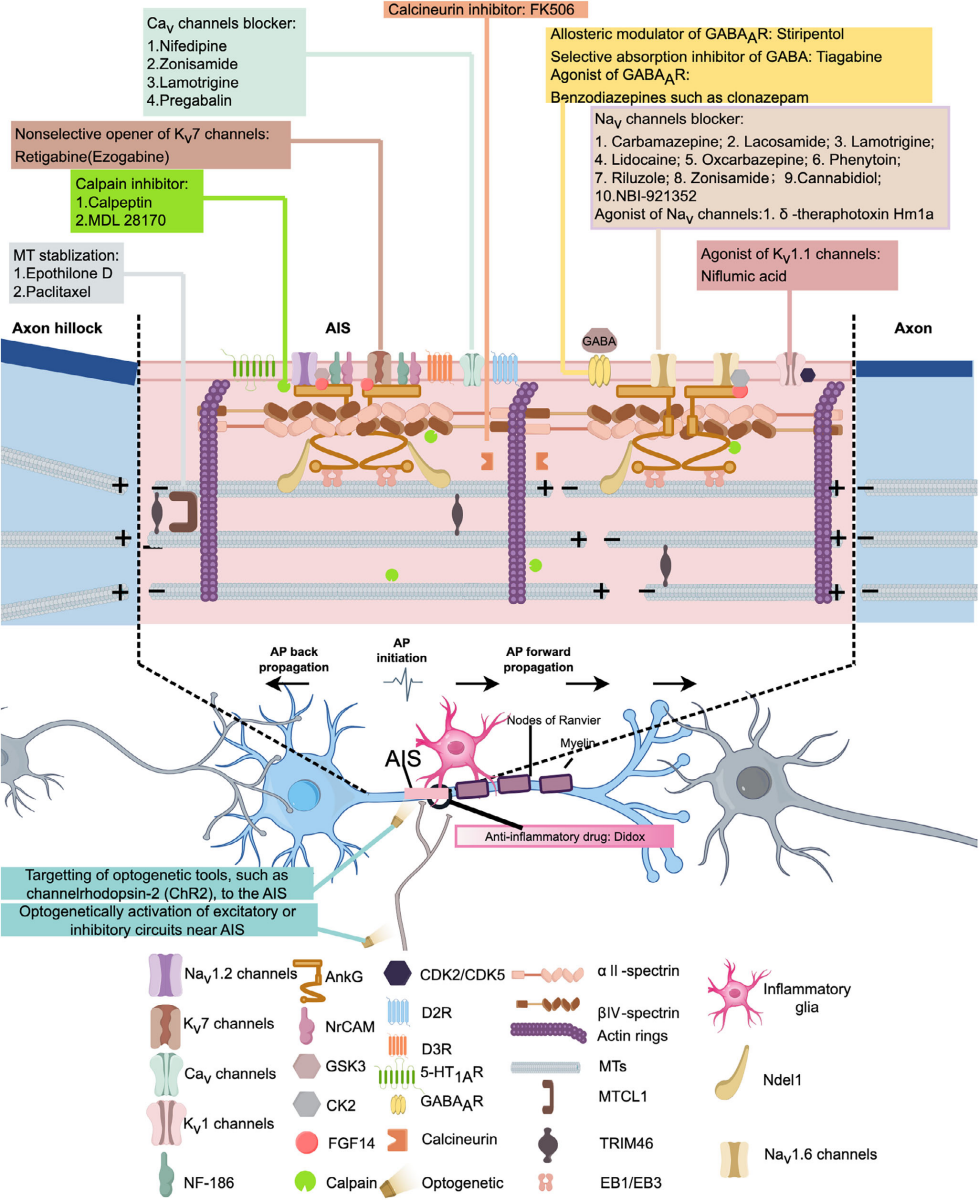
Therapeutic strategies targeting the AIS for neurological disorders. Ion channel inhibition or activation has emerged as a key therapeutic strategy for treating various neurological disorders. Additional pharmacological targets include GABAergic synapse stabilization, MT stabilization, calpain inhibition, and anti‐inflammatory mechanisms. Furthermore, optogenetic modulation techniques targeting the AIS through light stimulation, along with neuronal circuits adjacent to the AIS, are emerging as potential future therapeutic approaches.

Ion channels at the AIS play a central role in the pathogenesis of numerous neurological and psychiatric disorders. Although they are not specifically distributed at the AIS, due to their high‐density distribution at the AIS, they are also the most promising therapeutic targets. Similarly, pathological studies have also confirmed their potential. After TBI, Na_V_1.6 aggregates disperse [235]. In AD mouse models, Na_V_1.6 suppression has been proven to rescue cognitive deficits and hyperexcitability [220]. In ALS, its expression is also altered in a stage‐specific manner [12]. *SCN2A* is a risk gene for epilepsy [59, 65], schizophrenia [60], BD [74], and ASD [232]. *SCN1A* mutations are common in epilepsy and ASD [53, 65, 223]. Furthermore, Na_V_1.7 concentration at the AIS directly mediates neuropathic pain. Mutations in *KCNT1* (Slack channel) cause AIS elongation and upregulation of Na_V_ channels in epilepsy [225]. Similarly, K_V_7.3 mutations in ASD [70] and altered methylation in BD highlight its role [71]. Dysregulation of K_V_3.1 and K_V_2.1 is also respectively associated with schizophrenia and BD [68, 74]. The therapeutic potential of these ion channels has been further confirmed by existing clinical drugs, but more specific drugs are still needed. One major challenge in pursuing this strategy is that many voltage‐gated ion channels are ubiquitous in peripheral tissues, such as muscle and heart. This requires the development of drugs with selectivity for brain‐specific isoforms. The greater challenge lies in achieving subcellular specificity for the AIS itself. This goal remains an important frontier in neuropharmacology, due to the structural limitations and molecular complexity of this compartment. The US FDA‐approved drug riluzole, a TTX‐sensitive sodium channel blocker, has been demonstrated effective in ALS treatment [94]. Similarly, Na_V_ channel modulators such as phenytoin, carbamazepine, and lamotrigine remain mainstays of epilepsy treatment [237]. Notably, both lamotrigine and TTX show potential for AD intervention [220]. Beyond Na_V_ channels, the K_V_7.2 channel activator ezogabine (retigabine) has been approved for the treatment of partial epilepsy in adults [238]. Based on this, several novel candidates targeting different subtypes of ion channels have shown promise in preclinical models. The spider venom‐derived Hm1a peptide enhances Na_V_1.1 currents and rescues seizure phenotypes in Dravet syndrome mice [239, 240]. Cannabidiol inhibits Na_V_1.2‐mediated resurgent Na^+^ currents implicated in neuronal bursting [241]. And the selective Na_V_1.6 inhibitor NBI‐921352 suppresses seizures in *SCN8A*‐related epilepsy mouse [242, 243] (Figure 6).

### 6.2 Harnessing AIS Plasticity for Circuit Repair

Structural plasticity of the AIS is a fundamental mechanism for regulating neuronal excitability. AIS plasticity changes in response to variations in synaptic input, neural activity, or injury [244], thereby regulating the neuronal input–output [144]. These activity‐dependent changes ensure proper balance of excitation/inhibition (E/I) within neural networks, and can be regarded as part of a compensatory mechanism for maintaining network activity within a physiological range [109, 140]. Therefore, targeting AIS plasticity and repairing specific neural circuits represents an emerging therapeutic avenue.

A study has demonstrated that AIS plasticity is related to a simple form of motor learning. In spinal motoneurons of rats, a longer AIS length is associated with successful H‐reflex up‐conditioning, and the increased length correlates with greater H‐reflex increase. In contrast, increased GABAergic terminals at the AIS and reduced AnkG immunoreactivity are associated with successful down‐conditioning, and increased GABAergic terminals and reduced AnkG immunoreactivity correlate with greater H‐reflex decrease. However, the length or location of the AIS does not correlate with successful down‐conditioning. These results indicate that AIS plasticity is specifically associated with this form of motor learning and may contribute to it, suggesting its potential as a therapeutic target for restoring motor learning behavior after nerve injury [245].

A highly specific pathway for modulating the AIS is the special inhibitory input from ChCs. These ChCs specifically signal to the AIS of local PyNs, regulating AP initiation and neuronal excitability, thereby influencing network operations and advanced cognitive functions such as working memory [183, 185, 186, 187]. Critically, targeted manipulation of ChCs alone is sufficient to induce homeostatic changes at the AIS, including both structural modifications and alterations in Na_V_1 channel expression at the AIS. These changes subsequently lead to shifts in AP threshold and neuronal excitability. Importantly, the timing of this ChC‐induced AIS plasticity coincides with the recovery of behavioral defects. This indicates that AIS plasticity changes triggered by ChCs inputs can have physiological consequences [181, 182]. These findings provide a solid basis for boosting axo‐axonic inhibition efficacy and specificity as a promising therapeutic target for normalizing neural circuit output in neurological disorders.

Noninvasive neuromodulation represents another promising strategy for inducing therapeutic AIS plasticity. Research demonstrates that long‐term static magnetic stimulation (SMS) can trigger structural AIS plasticity in cortical neurons. A 6‐h exposure results in shortened AIS, while a 48‐h exposure causes a distal shift in AIS position. And these structural changes persist for at least 24 h after stimulation ends [246]. SMS can also modulate corticospinal excitability and motor behaviors. These findings indicate that SMS is a feasible tool for externally inducing persistent AIS remodeling, providing a foundation for the development of novel therapeutic strategies targeting the restoration of neural circuit activity in neurological disorders.

Despite its considerable therapeutic potential, targeting AIS plasticity still faces numerous challenges. Excessive or maladaptive AIS plasticity can lead to hyperexcitability and pathology, as observed in epilepsy. Therefore, how to precisely fine‐tune AIS properties to restore the delicate E/I balance within neural networks is the major focus of future research.

## 7 Experimental and Clinical Assessment Tools

In recent years, a series of methodological innovations and breakthroughs have deepened our understanding of the basic biology of AIS in neurodevelopment and neurological diseases. This section systematically reviews these key research techniques and methods.

### 7.1 Live Imaging Tools

Traditionally, investigation of AIS architecture has mainly relied on immunofluorescence techniques applied to fixed tissues. In recent years, the emergence of live‐cell imaging methodologies, including antibodies targeting the extracellular domain of NF‐186 and the method of transfecting AnkG–GFP constructs [17, 247], has enabled us to observe the AIS in live neurons in real‐time, deepening our understanding of the dynamic alterations of AIS in physiological and pathological states.

The live‐imaging tools are crucial for monitoring dynamic AIS plasticity. A systematic evaluation was conducted on five labeling approaches for DGCs in dissociated hippocampal cultures. The results indicate that each method has its own unique limitations and advantages. The antibody labeling method using the neurofascin antibody can accurately capture the baseline AIS structure, but fails to rapidly track its activity‐dependent changes [248]. All three full‐length protein fusions have their own drawbacks: NF‐186–GFP and Na_V_β4–GFP failed to precisely localize to the AIS, while the overexpressed 270kDa‐AnkG–GFP leads to abnormal AIS elongation. The genetically encoded YFP‐Na_V_II–III probe has significant advantages [248]. The intracellular loop between Na_V_ transmembrane domains II and III (Na_V_II–III) is a necessary domain for these channels to bind AnkG and localize to the AIS. Studies have shown that it can accurately report long‐term (48 h) activity‐dependent changes in AIS position [78, 137, 249], and has minimal perturbation with neuronal excitability. It also has certain drawbacks, during activity‐dependent shortening events, its distribution changes are relatively slower compared with endogenous proteins. However, compared with the other four methods, its specificity makes it more suitable for long‐term observation of AIS plasticity both in vitro and in vivo [248].

Recently, a novel knock‐in mouse model has brought a new breakthrough in observing AIS in vivo [19]. This mouse expresses the AnkG–GFP fusion protein in a cre‐dependent manner from the native *ANK3* locus [19]. Crucially, the GFP tag does not disrupt AIS structural integrity and can maintain normal electrophysiological properties, and thus does not alter neuronal excitability. Combined with chronic cranial window imaging, the same AIS can be tracked over days of PyN in the somatosensory cortex. Under baseline conditions, the AIS structure exhibited significant stability [19]. A key breakthrough of this model is its ability to observe the rapid and bidirectional AIS plasticity in real‐time. By applying high potassium to a single neuron of the acute brain slices from these mice, the proximal AIS shortened within 10 min and is reversible [19]. Furthermore, AIS can be labeled and imaged in multiple brain regions, such as the superficial cortical layers and the basolateral amygdala [19]. This powerful mouse model offers the possibility of studying AIS dynamics in vivo under both physiological and pathological conditions.

### 7.2 High‐Resolution Imaging of the AIS

Super‐resolution stimulated emission depletion (STED) microscopy has significantly deepened our understanding of AIS nanoscale architecture. STED imaging reveals that the periodic actin structure is present in AIS, and the actin patches are colocalized with pre‐synaptic markers [17]. Notably, STED precisely shows the ∼190 nm periodic colocalization of K^+^ leak channels TRAAK with AnkG, demonstrating TRAAK integrated into the βIV‐spectrin‐based cytoskeletal [102].

Stochastic optical reconstruction microscopy (STORM) can quantitatively measure the AIS nanoscale architecture. First, this technique reveals that the ∼190 nm periodic lattice is formed by longitudinal head‐to‐head βIV‐spectrin subunits connecting with submembrane actin rings [16]. Furthermore, it reveals clustered distributions of NF‐186 and Na_V_ channels along the periodic βIV‐spectrin bands [16]. Additionally, multicolor 3D‐STORM imaging shows that the N‐terminus of AnkG binds to βIV‐spectrin and membrane proteins in the submembrane lattice, while the C‐terminus of AnkG extends radially toward the axoplasm [16]. And a 32 nm radial interval exists between spectrin‐binding domains and C‐terminal domains of AnkG [16]. The nano‐architecture of AIS serves as an important foundation for its resistance to cytoskeletal perturbations.

Platinum‐replica electron microscopy (PREM) has played a significant role in revealing AIS molecular architecture during neuronal development. Through PREM, it has been confirmed that AIS assembly progresses from MT bundling to the formation of a dense fibrillar‐globular coat [22]. When combined with optical super‐resolution microscopy, PREM reveals that the actin ring in a braided structure—approximately 18.2 ± 0.3 nm in diameter—consists of two elongated and intertwined actin filaments interconnected with a tightly packed array of arranged spectrins [18]. The PREM further reveals that AnkG connects the periodic structure with MTs through its C‐terminus [18]. Furthermore, combining PREM with super‐resolution technology discover that clathrin‐coated pits (CCPs) with characteristic honeycomb patterns exist in the AIS. And by using it together with structured illumination microscopy reveal that CCPs localize within spectrin mesh‐free areas. Furthermore, it discovers that AnkG is distributed throughout the spectrin mesh, including areas adjacent to CCP clearings [29].

Live‐cell single‐molecule imaging combined with the HaloTag knock‐in technology with bright Janelia Fluor dyes can be used to observe the dynamic localization of AIS proteins. This technology enables nanoscale tracking of Na_V_1.2 and Na_V_1.6 with high temporal resolution [84]. The methodology not only preserves native protein distribution patterns, but also enables the control of labeling density via the pulse‐chase method, thereby capturing long trajectories of trafficking proteins [84].

### 7.3 Proteome of the AIS

For a long time, due to AIS's structural properties and technical limitations, proteome analysis of AIS has been extremely difficult. There are mainly three reasons: (1) many AIS proteins are tightly bound to AnkG‐dependent cytoskeleton resulting in insolubility in detergents, thus traditional immunoaffinity methods cannot effectively purify them [250]; (2) second, many AIS proteins are not limited to AIS or enriched at the AIS [250]; (3) as a microdomain on the axon, AIS is extremely difficult to separate [250].

Recent studies have shown that the combination of enzyme‐mediated proximity‐dependent biotinylation with quantitative mass spectrometry has enabled comprehensive profiling of the AIS proteome of hippocampal neurons in vitro [134, 250]. This method is implemented by generating BirA* fusion proteins with three different localized AIS proteins: NF‐186, Ndel1, and TRIM46. These three proteins mark different AIS domains, successfully capturing the core AIS components and identifying numerous novel AIS proteins [250]. NF‐186 marks the plasma membrane‐proximal region, Ndel1 marks the cytoplasmic region, and TRIM46 marks the MT‐associated compartments [250]. However, this method still has certain limitations. It fails to detect some known proteins, such as Na_V_1.6, KCNQ2/3, NrCAM, PSD‐93, and Caspr2 [250]. This may result from the spatial limitations of the BirA* fusion site or the developmental time constraints in capturing these interactions [250].

The Selective Proteomic Proximity Labeling Assay Using Tyramide, also known as the Biotinylation by Antibody Recognition methodology [119], overcomes these limitations by employing the antibody‐directed proximity biotinylation technique [119]. This approach utilizes a specific antibody targeting the extracellular domain of neurofascin to direct the HRP‐conjugated secondary antibodies to the AIS [119]. In the presence of biotin‐tyramide and hydrogen peroxide, biotin phenoxyl radicals are produced. These radicals can label membrane proteins within a range of ∼250 nm [119]. Using this strategy in live neurons at multiple developmental timepoints, the known AIS extracellular components and numerous novel membrane proteins with unique temporal enrichment patterns are successfully identified [119]. Furthermore, the newly developed immunoproximity labeling for AIS (IPL‐AIS) methodology, which targets the endogenous proteins of AIS with antibodies against NF‐186, has achieved an amplification labeling radius of 250–500 nm [134]. Notably, this method has also captured the dynamic developmental changes of the AIS proteome from DIV7 to DIV21. This indicates that IPL‐AIS is a powerful tool that can be used to define AIS composition and assembly mechanisms [134].

### 7.4 Electrophysiological Recordings From the AIS

AIS is the main site for AP initiation, a function that can be studied through electrophysiological recordings [64, 77, 111]. In PyN of rat PFC slices, recording axonal blebs at different distances from the soma can resolve the spatial segregation of Na_V_ channel subtypes. Proximal blebs (<40 µm) likely reflect both Na_V_1.2 and Na_V_1.6 activity, while distal blebs (>70 µm) primarily reflect Na_V_1.6 activity [156]. A deeper understanding of AIS function comes from the analysis of AP waveform derivatives. The first (d*V*/d*t*) and second (d*V* ^2^/d*t* ^2^) derivatives of APs can distinguish AIS‐initiated spikes [82, 83]. This approach can detect impairments in spike initiation and backpropagation caused by altered AIS‐localized ion channels distribution [83]. These electrophysiological recordings provide a direct experimental approach for studying the crucial role of the AIS in influencing neuronal excitability through AP initiation.

### 7.5 Promising Clinical Tools

Currently, it is impossible to directly observe AIS changes in patients. Some existing research methods provide a foundation for future diagnostic approaches. Postmortem studies remain crucial for validation. For instance, immunohistochemistry shows that at the AIS of patients with schizophrenia, the level of GAT‐1‐positive ChC cartridges decreases, while the level of GABA_A_ receptor α2 subunits increases [201, 251]. Genetically, testing genes encoding critical AIS proteins provides certain predictive value. Identifying pathogenic variants in *SCN2A* helps predict the risk of early‐infantile epileptic encephalopathies [84, 252]. Specific polymorphisms in *ANK3* are related to a high risk for BD [228]. Although these genes are not exclusively expressed at the AIS, testing them serves as a key alternative indicator for evaluating potential AIS dysfunction. Electroencephalography can serve as a promising auxiliary tool for observing the functional consequences of impaired AIS‐related disorders. For example, it can detect generalized spike and slow waves associated with Dravet syndrome [253]. These complementary methods provide the foundation for future indirect clinical assessments of pathological changes caused by impaired AIS integrity.

## 8 Future Directions and Knowledge Gaps

### 8.1 The “Chicken or Egg” Question: Causality in AIS Pathology

A core unresolved issue is whether AIS alterations are a primary cause of neural circuit dysfunction or a secondary consequence of widespread neuropathology. A large amount of evidence from neurodegenerative and neuropsychiatric disorders indicates AIS impairment as an early and potentially causal event in disease pathogenesis. In AD, APP accumulation shortens AIS independently of Aβ, indicating that AIS is one of the direct targets in the early stage of disease [11]. In ALS, TDP‐43 aggregation in the cytoplasm induces reversible structural remodeling of AIS [221]. In the early stage, AIS elongates, while in the later stage, it shortens, accompanied by specific channelopathies [12]. This suggests that AIS dysfunction is an indispensable part of the pathological process, rather than a passive byproduct. In epilepsy, numerous causative genes encode core AIS components such as *SPTAN1* and *SCN2A* [59, 212]. Most convincingly, in a *15q13.3* microdeletion epilepsy model, restoration of AnkG expression can reverse pathological phenotypes, providing direct evidence that AIS defects can serve as the critical driver for disease pathology [226]. These findings suggest that AIS dysfunction may serve as a common pathogenic mechanism in various neurological and psychiatric disorders. Future research should focus on developing measures for temporally controlled and cell‐type‐specific interventions to directly manipulate AIS components (e.g., ion channels, AnkG) in vivo, determining whether specific AIS perturbations are sufficient to induce disease‐like pathology, and whether correcting these perturbations can restore functional integrity.

### 8.2 The Targeting Challenge: From Observation to Therapeutic Intervention

A central challenge in translating AIS research is the development of strategies that can selectively modulate this domain while sparing other neuronal domains. Current optogenetic tools for AIS, such as those using the AnkG‐binding domains of Na_V_1.2 (ChR2–YFP–Na_V_II–III), Na_V_1.6 (ChR2–GFP–Na_V_II–III), or ankyrin–hChR2 [95, 96, 97], are all facing these limitations. The method based on intercellular loop fragments often acts as a competitive inhibitor, replacing endogenous Na^+^ and K^+^ channels and damaging normal firing properties [95, 96, 97]. Notably, ChC has specific inhibitory inputs to the AIS of local PyN [178, 182, 187, 189]. Inspired by this biological specificity, we propose two strategic directions: first, we could aim to design novel targeting tools by utilizing the specific molecular interface of the ChC–AIS synapse [179, 193]; second, seeking circuit‐based strategies to manipulate ChC may indirectly regulate AIS excitability [185]. Given its specificity, the ChC microcircuit emerges as a promising therapeutic target for restoring AIS function under pathological conditions.

Recent advances, such as the AnkG–GFP knock‐in mouse model, enable noninvasive AIS imaging in vivo, overcoming prior limitations in AIS‐specific genetic labeling [19]. Another challenge in the future is the integration of AIS‐specific proteomic datasets to identify truly unique molecular characteristics, such as AIS‐enriched receptor subtypes, phosphorylation codes, or protein isoforms that are functionally distinct from somatodendritic counterparts. By targeting these signatures, AIS excitability could be fine‐tuned without off‐compartment effects. Therapeutic development may harness AIS‐specific regulatory elements (e.g., Ank3 promoters) for cell‐type‐selective interventions, including gene therapy or CRISPR‐based editing. Together, these approaches promise to bridge the gap between AIS biology and targeted neuromodulation, opening new avenues for treating neurodevelopmental and neurological disorders.

## 9 Conclusion

### 9.1 Summary of AIS Roles in Physiology and Pathology

In this review, we summarize the extensive AIS biological functions, covering aspects from maintaining neuronal polarity, initiating APs, to serving as a dynamic and plastic subdomain for homeostatic regulation [3, 4, 9, 10]. We elaborate on its unique molecular architecture—centered on the scaffold protein AnkG—that coordinates a complex of cytoskeletal proteins, ion channels, and cell adhesion molecules to execute these functions [7]. Crucially, across a wide range of neurological disorders, such as AD, ALS, epilepsy, and schizophrenia, the plasticity and structural integrity of the AIS are frequently compromised, manifested as alterations in its structure, molecular composition, and ion channel density [11, 12, 13, 14, 15]. Notably, a growing body of evidence suggests that these changes are not merely secondary consequences of pathology but may act as core drivers of neuronal hyperexcitability in the disease process [11, 12].

### 9.2 Challenges and Future Directions in Mechanistic Understanding

Translating these findings into clinical applications requires overcoming numerous significant obstacles. A primary challenge lies in resolving the “chicken‐or‐egg” issue of causality in diseases involving AIS alterations. Future research should utilize cell‐type‐specific in vivo models to systematically determine which neuronal populations exhibit AIS vulnerability [254]. Spatial mapping resources, such as the Allen Brain Atlas, can help identify AIS‐injured neuronal circuits, AIS‐associated ligand–receptor pairs, and AIS‐related genetic variations. This will provide a foundation for the development of transgenic tools targeting specific AIS subtypes and the design of functional optogenetic tools [134, 255]. Simultaneously, by integrating animal experiments with multiomics data from human biofluids and postmortem tissues, it is possible to further clarify the core pathogenic pathways and identify the most promising therapeutic intervention targets.

### 9.3 Technological Convergence and Therapeutic Horizons

Continuous technological innovation is conducive to the progress of AIS research and its clinical translation. The convergence of super‐resolution imaging, patch‐seq, in vivo biosensors, and highly specific proteomics techniques (such as IPL‐AIS) provides insight into the nanoscale dynamics of AIS under physiological and pathological conditions, alterations in AIS spike properties, and molecular alterations [134, 255]. From a therapeutic perspective, two directions hold particular promises: first, the development of novel targeting strategies for specific ChC–PyN synapse [181, 182]; and second, the direct targeting of AIS‐associated proteins, such as specific ion channels to modulate neuronal excitability, or the scaffold protein AnkG to stabilize its structure, while retaining the core physiological functions of AIS [239, 242].

### 9.4 Concluding Perspective: The AIS as a Nexus of Disease Mechanisms and Therapeutic Innovation

In conclusion, an increasing amount of evidence clearly indicates that AIS is not only a critical locus in the pathogenesis of neurological diseases but also a source with immense potential for transformation. The detection of AIS‐related components in biofluids—such as anti NF‐186 [256] antibodies in CNS and PNS disorders, or spectrin breakdown products enriched in CSF vesicles after TBI [257]—provides a convincing path to developing biomarkers of AIS integrity. Coupling these biomarkers with large‐scale human brain transcriptomic atlases further enables AI‐driven pathology prediction and mechanistic stratification, heralding a new paradigm in disease classification [258, 259, 260, 261]. Beyond diagnostics, the AIS represents a novel and compelling therapeutic target for modulating neural circuit synchrony. Deciphering the molecular logic of the AIS is therefore paramount to advancing the field from syndromic diagnosis toward a precise, mechanism‐based understanding and treatment of neurological disorders.

## Author Contributions

Conceptualization: Dong‐Yan Song, Lin Yuan, Weiguo Yang, Wen Li, and Jia‐Yi Li. Funding acquisition: Weiguo Yang, Wen Li, and Jia‐Yi Li. Supervision: Wen Li and Jia‐Yi Li. Visualization: Dong‐Yan Song. Writing – original draft: Dong‐Yan Song. Writing – review and editing: Lin Yuan, Weiguo Yang, Wen Li, and Jia‐Yi Li. All the authors have read and approved the final manuscript.

## Funding

This work was supported by grants from the National Natural Science Foundation of China (No. 82361138574, 82371273, 81430025, 31800898 and 32471087), the Swedish Research Council (2023‐02216), the Strategic Research Area Multipark (Multidisciplinary research in Parkinson's disease at Lund University, Sweden), the Department of Science and Technology of Liaoning Province (2024JH6/100800008, 2025JH2/102800060), and the Department of Education of Liaoning Province (LJKMZ20221207).

## Ethics Statement

The authors have nothing to report.

## Conflicts of Interest

The authors declare no conflicts of interest.

## Data Availability

All data needed to evaluate the conclusions in the paper are present in the paper.
